# Improving preeclampsia risk prediction by modeling pregnancy trajectories from routinely collected electronic medical record data

**DOI:** 10.1038/s41746-022-00612-x

**Published:** 2022-06-06

**Authors:** Shilong Li, Zichen Wang, Luciana A. Vieira, Amanda B. Zheutlin, Boshu Ru, Emilio Schadt, Pei Wang, Alan B. Copperman, Joanne L. Stone, Susan J. Gross, Yu-Han Kao, Yan Kwan Lau, Siobhan M. Dolan, Eric E. Schadt, Li Li

**Affiliations:** 1 Sema4, Stamford, CT USA; 2 Department of Obstetrics, Gynecology, and Reproductive Science, Icahn School of Medicine at Mount Sinai, New York, NY USA; 3 Department of Genetics and Genomic Sciences, The Icahn Institute for Genomics and Multiscale Biology, Icahn School of Medicine at Mount Sinai, New York, NY USA; 4 Reproductive Endocrinology and Infertility, Reproductive Medicine associates of New York, New York, NY USA

**Keywords:** Pregnancy outcome, Risk factors

## Abstract

Preeclampsia is a heterogeneous and complex disease associated with rising morbidity and mortality in pregnant women and newborns in the US. Early recognition of patients at risk is a pressing clinical need to reduce the risk of adverse outcomes. We assessed whether information routinely collected in electronic medical records (EMR) could enhance the prediction of preeclampsia risk beyond what is achieved in standard of care assessments. We developed a digital phenotyping algorithm to curate 108,557 pregnancies from EMRs across the Mount Sinai Health System, accurately reconstructing pregnancy journeys and normalizing these journeys across different hospital EMR systems. We then applied machine learning approaches to a training dataset (*N* = 60,879) to construct predictive models of preeclampsia across three major pregnancy time periods (ante-, intra-, and postpartum). The resulting models predicted preeclampsia with high accuracy across the different pregnancy periods, with areas under the receiver operating characteristic curves (AUC) of 0.92, 0.82, and 0.89 at 37 gestational weeks, intrapartum and postpartum, respectively. We observed comparable performance in two independent patient cohorts. While our machine learning approach identified known risk factors of preeclampsia (such as blood pressure, weight, and maternal age), it also identified other potential risk factors, such as complete blood count related characteristics for the antepartum period. Our model not only has utility for earlier identification of patients at risk for preeclampsia, but given the prediction accuracy exceeds what is currently achieved in clinical practice, our model provides a path for promoting personalized precision therapeutic strategies for patients at risk.

## Introduction

Preeclampsia (PE) remains one of the great challenges in obstetrics. It contributes substantially to maternal morbidity and maternal mortality worldwide, and within the US, accounted for 6.9% of pregnancy-related deaths from 2011 to 2016 (CDC Reproductive Health: Maternal Mortality) and is substantially higher in other regions. There are significant implications for newborns as well, with PE being responsible for a large percentage of medically indicated preterm deliveries^1^.

PE is characterized by elevated blood pressure during pregnancy, starting after 20 gestational weeks. While moderately elevated blood pressure itself is not necessarily harmful, in the case of PE, elevated blood pressure reflects the multi-system endothelial dysfunction leading to vascular, renal, and liver impairment associated with this disease. Eclampsia, defined as convulsions during pregnancy and/or postpartum irrespective of hypertension, is an especially devastating outcome and may be associated with maternal hypoxia and death. The underlying mechanisms are not fully understood but recent evidence suggests involvement of multiple factors and pathways, including maternal factors and abnormal trophoblast differentiation^2^. This underlying complexity helps to explain the unpredictable nature of PE. PE can vary not only in severity, but also in timing of onset and impact on fetal growth. Although there are serious clinical sequela due to PE, antenatal monitoring to determine when delivery outweighs the risk of ongoing expectant management delivery is the standard clinical care plan for PE patients, given delivery is currently the only recognized treatment for PE.

Currently, women are routinely screened for PE at the first prenatal visit using clinical factors. Some centers may also include serum protein markers and ultrasound Doppler studies to screen for early PE. During subsequent visits, blood pressure and proteinuria screening are conducted. Ideally, improved screening could direct clinical care through increased prenatal surveillance and adoption of prophylactic measures, such as low dose aspirin that has been shown to reduce risk of preterm PE and potentially other perinatal complications^3^ (ACOG Committee Opinion No. 743; USPSTF, 2017). In addition, accurate identification of risk could allow for escalation to a higher level of care facility for delivery. However, there is still a lot of room for improvement with respect to PE screening. The genome, transcriptome, proteome, and metabolome have all been interrogated and have generated some promising data^4–9^. However, currently there are no omics-based biomarkers available for clinical use. Furthermore, all the current screening methodologies focus on a relatively small number of maternal characteristics, and usually just one time point at early pregnancy that remains the same over the course of gestation^10^. Considering the number of prenatal visits that occur over a well-defined time range, there remains an unmet need for longitudinal PE assessment at each encounter that accounts for changes in clinical measurements within an individual’s characteristics throughout pregnancy. Further, new onset preeclampsia can occur postpartum^11^ and remains a common reason for postpartum readmission^12^ highlighting a need for ongoing risk assessment intrapartum and in the early postpartum period. With PE rates rising along with maternal mortality in the U.S^13^., a more robust approach that can predict antenatal, intrapartum, and postpartum PE is still very much needed.

To the best of our knowledge, large-scale EMR data have not been systematically mined to identify novel features associated with PE risk and to model these data using machine learning approaches to establish whether this wealth of longitudinal, high-dimensional patient-level data contained in EMRs can improve PE risk prediction. The increasing accessibility of large-scale EMR data integrates laboratory-based molecular and biochemical tests, disease diagnoses, procedures, and prescriptions, along with outcomes during the pregnancy journey. Further, abstracting patient journey information from these records, normalizing the data across systems, reconstructing pregnancy journeys, and modeling these journeys using state-of the-art data analytic approaches that can account for dynamic state changes provides for the potential to better model PE risk through the course of pregnancy, compared to what is achieved in today’s standard of care.

Here we build predictive models from digitally reconstructed pregnancy journeys derived from the EMR data from the Mount Sinai Health System (MSHS) in New York City, among the largest and most diverse health systems in the U.S., to assess the risk of PE across 17 time points throughout the antepartum, as well as intrapartum and postpartum periods of pregnancy. Appropriately curated pregnancy journeys derived from EMR data provide a more expansive, feature-rich context in which to study the pathophysiology of PE towards constructing more predictive models to identify patients at risk for PE. After identifying 83,954 patients with pregnancies represented in the MSHS EMR, we reconstructed the full longitudinal health course through these pregnancies (referred to as pregnancy journeys) using a pregnancy journey construction algorithm resulting in the identification of 80,021 patients in which 108,557 complete pregnancy journeys were captured by the EMR. We then developed a digital PE phenotyping rules-based algorithm based on clinical criteria established by the American College of Obstetricians and Gynecologists (ACOG)^14^ to identify patients diagnosed with PE at different periods of their pregnancy. With complete pregnancy journey information and the PE diagnosis labels, we constructed predictive models at 19 different time points across the three major pregnancy time periods (ante-, intra-, and postpartum) by applying state-of-the-art statistical and machine learning methods to data collected for patients throughout their pregnancy journey. We validated the predictive models we trained using data from one hospital within the MSHS, and another independent dataset derived from other hospitals within the MSHS. Our PE risk assessment model could be applied in clinical practice by extracting the relevant input features for the model from the patients’ electronic medical records and running the model on those data. Furthermore, using different approaches to interpret predictions, we reveal the connections between clinical features and PE risk to help understand the potential research areas for exploring pathophysiology of PE.

## Results

### Reconstructing pregnancy journeys from electronic medical record data

One of the limitations of current-day EMR systems in widespread use is that they do not naturally capture and represent patient journeys through specific episodes through a patient’s health course. EMR systems are transactional, automating the capturing of a patient visit and recording of the clinical measures, labs, procedures, and prescriptions generated on a patient over the course of their visit. Most EMRs in widespread use are not set up to provide a longitudinal view of a patient along a particular health course journey such as pregnancy with all the corresponding data generated on the patient over that journey. Thus, we developed a pregnancy journey construction algorithm to identify 83,954 patients with 114,312 pregnancies represented in the MSHS EMR systems and to reconstruct 108,557 full pregnancy journeys of 80,021 patients between 2002 and 2019 (see Methods section).

### Patient characteristics across a training and two independent test datasets

**Fig. 1:**
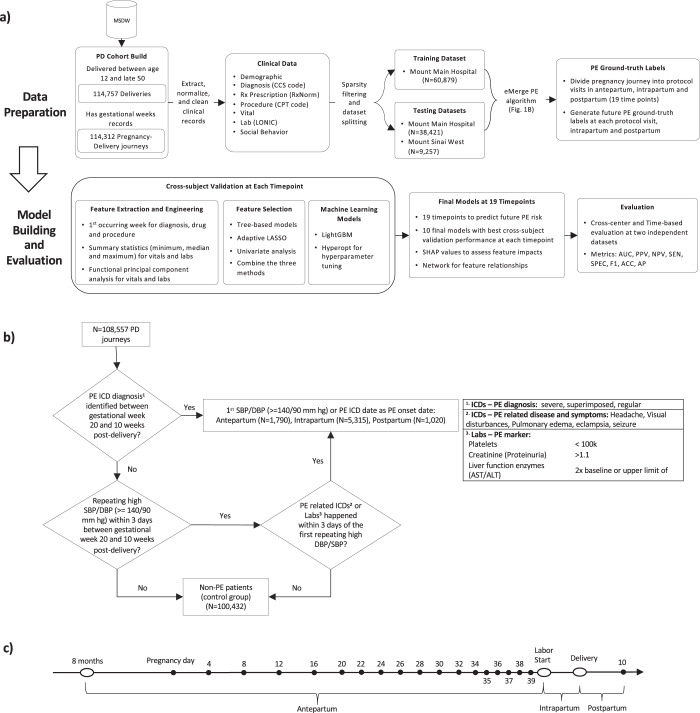
Overview of study design and model development. **a** The workflow of the study outlines the cohort construction, patient characteristics extraction, dataset splitting into training and testing datasets (including subdivision into antepartum, intrapartum, and postpartum), feature engineering, feature selection, machine learning models (cross-subject validation) and final evaluation. **b** The proposed eMerge algorithm to identify preeclampsia (PE) patients to construct the binary prediction problem. **c** The schematic of 19 timeline models including: monthly models, weeks 4–20; biweekly models, weeks 22–34; weekly models, weeks 35–39; intrapartum and postpartum model.

We retrieved all relevant clinical characteristics on the patients in this dataset, including demographics, diagnoses, drug prescriptions, procedures, vital signs, and lab values (Fig. 1a). In total we captured 3230, 4136, and 5391 clinical features for ante-, intra- and postpartum, respectively, represented in the EMR on these patients and 46,725,028 data points overall, providing among the most comprehensive datasets available in the context of the pregnancy journey, to take a more data-driven approach to evaluating PE risk. Women delivered at one of two main inpatient facilities, Mount Sinai Hospital (MSH) and Mount Sinai West (MSW). We split patient journeys collected from MSH into a training set (*N* = 60,879) and a test set (*N* = 38,421) irrespective of the timing, and we used MSW (*N* = 9257) from a different geographic region in NYC as a second test set.

To identify patients diagnosed with PE during the course of their pregnancy from these datasets, we developed and applied a rule-based digital phenotyping algorithm (Fig. 1b) to identify 5663 (9.3%) PE patients from the 60,879 patients in the training dataset. We further identified 2064 (5.4%) PE patients from the MSH test dataset and 398 (4.3%) PE patients from the MSW test dataset, respectively. The PE prevalence observed across the various datasets is consistent with prior published literature: 2–8% in the general population^15^.

Patient demographics and characteristics collected 8 months prior to pregnancy as baseline were significantly different between the MSH training set, the MSH test set, and the MSW test set, indicating differences in regional geographic and socioeconomic status, and shifting demographics over time. More detailed summaries of the characteristics of these different datasets are provided in Table 1, where we note statistically significant differences with respect to Medicaid rates, population composition, and average pregnancy ages, among several other features, between the different datasets.

**Table 1:** Characteristics of patients in MSH training dataset, and MSH and MSW test dataset.

Cohorts	Mount Sinai Hospital (MSH) training set	Mount Sinai Hospital (MSH) test set	Mount Sinai West/UW/BI/SL test set	*P*-value	Test
Features at Baseline	60879	38421	9257		
Age at pregnancy, median [Q1,Q3]	31.0 [26.0,35.0]	31.0 [27.0,35.0]	33.0 [29.0,36.0]	<0.001	Kruskal–Wallis
Weight (kg), median [Q1,Q3]	63.5 [56.0,74.4]	63.5 [56.2,74.4]	63.0 [56.2,72.6]	0.084	Kruskal–Wallis
Height (cm), median [Q1,Q3]	162.6 [157.5,167.6]	162.6 [157.5,167.6]	163.8 [160.0,168.9]	<0.001	Kruskal–Wallis
BMI (kg/m^2^), median [Q1,Q3]	23.8 [21.1,28.0]	24.0 [21.2,28.1]	23.2 [20.9,26.6]	<0.001	Kruskal–Wallis
SBP (mmHg), median [Q1,Q3]	110.0 [104.0,120.0]	112.0 [106.0,120.0]	112.5 [108.0,120.0]	<0.001	Kruskal–Wallis
DBP (mmHg), median [Q1,Q3]	67.5 [60.0,72.0]	69.0 [62.0,74.0]	70.0 [66.0,76.0]	<0.001	Kruskal–Wallis
**Race,** ***n*** **(%)**					
African American/Black	8336 (13.7)	3246 (8.4)	1399 (15.1)	<0.001	Chi-squared
Asian	4623 (7.6)	2754 (7.2)	1123 (12.1)		
Caucasian/White	32662 (53.7)	25686 (66.9)	4622 (49.9)		
Hispanic/Latino	9928 (16.3)	3253 (8.5)	280 (3.0)		
Multi-racial	589 (1.0)	208 (0.5)	110 (1.2)		
Native American	227 (0.4)	111 (0.3)	73 (0.8)		
Other	4112 (6.8)	2386 (6.2)	1090 (11.8)		
Unknown	402 (0.7)	777 (2.0)	560 (6.0)		
Medicaid, *n* (%)	22773 (37.4)	9956 (25.9)	1362 (14.7)	<0.001	Chi-squared
Miscarriage history, *n* (%)	1167 (1.9)	331 (0.9)	299 (3.2)	<0.001	Chi-squared
PE history, *n* (%)	350 (0.6)	140 (0.4)	28 (0.3)	<0.001	Chi-squared
Smoking history, *n* (%)	5876 (9.7)	2135 (5.6)	544 (5.9)	<0.001	Chi-squared
Alcohol use history, *n* (%)	8941 (14.7)	3902 (10.2)	546 (5.9)	<0.001	Chi-squared
**hospital,** ***n*** **(%)**					
Mount Sinai Hospital—Main Hospital	60879 (100.0)	38421 (100.0)		<0.001	Chi-squared
Mount Sinai West/UW/BI/SL			9257 (100.0)		
**PE type,** ***n*** **(%)**					
Antepartum	1213 (2.0)	437 (1.1)	140 (1.5)	<0.001	Chi-squared
Intrapartum	3787 (6.2)	1322 (3.4)	206 (2.2)		
None	55216 (90.7)	36357 (94.6)	8859 (95.7)		
Postpartum	663 (1.1)	305 (0.8)	52 (0.6)		
Pregnancy journey length (days), median [Q1,Q3]	273 [266,280]	275 [267,281]	277 [270,282]	<0.001	Kruskal–Wallis

### Performance of predictive model across pregnancy in training set

**Fig. 2:**
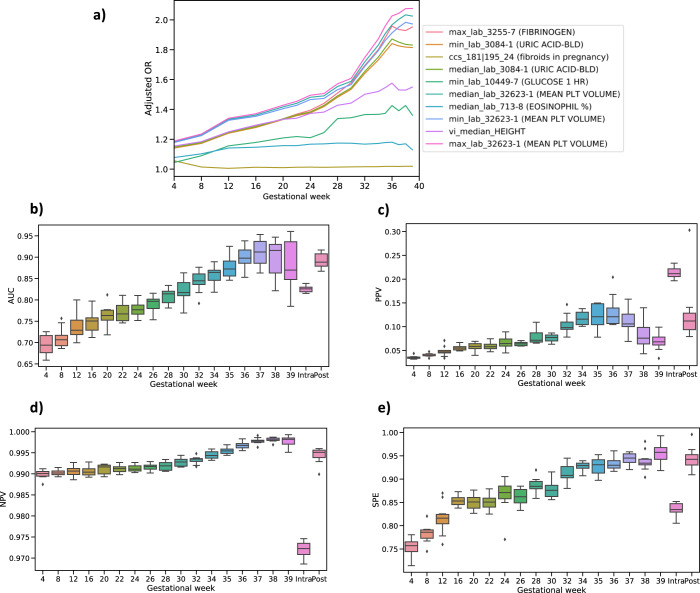
Model performance at different time points. **a** Features indicate different dynamical signals across the gestational weeks based on different adjusted odds ratio (OR). **b** Area under receiver operating characteristic curve (AUC) score for each time point. **c** Positive predictive value (PPV), along with preeclampsia risk in the population, at each time point. **d** Negative predictive value (NPV) at each time point. **e** Specificity (SPE) at each time point. The variation estimates were derived from 10-folds cross-subject validation from training set. For the box plots shown in **b**–**d**, the median, interquartile (1st and 3rd) range, and minimum and maximum values, are depicted by the center line, the bounds of the boxes, and the whiskers respectively.

In order to train predictive models for PE along the pregnancy journey, we divided the journey up into 19 time points that included dividing the antepartum period into 17 time points following a standard of care protocol for prenatal office visits at the participating site: 5 monthly visits spanning weeks 4–20, 7 biweekly visits spanning weeks 22–34, and 5 weekly visits spanning weeks 35–39;^16,17^ followed by intrapartum and postpartum periods as two independent time points with respect to the pathophysiology of PE (Fig. 1c). Given the large number of clinical features available from the EMR database for our datasets, for each of the 19 time points we employed several feature selection methods to choose features robustly that were consistently significantly different between patients diagnosed with PE and those without PE. Several features demonstrated consistently changing effects throughout the pregnancy (Fig. 2a), reinforcing the importance of partitioning the antepartum period into more granular time points to better isolate signals that may associate with the clinical manifestation of PE. For the monthly models (spanning weeks 4–20), our feature selection process identified between 19 and 36 unique features depending on the month; between 34 and 40 unique features for the biweekly models (weeks 22–34); and 35–40 unique features for the weekly models (weeks 35–39). We also selected 68 and 48 unique features, respectively, for the intrapartum and postpartum periods. All the selected unique features across the 19 time points are summarized in Supplementary Table 1–19. For each of the 19 time points, we trained gradient boosting models and tuned the parameters of these models using cross-subject validation. The cross-subject validation performance for each time point is summarized according to the area under the receiver operating characteristic curve (Fig. 2b; AUC), the positive predictive value (Fig. 2c; PPV), the negative predictive value (Fig. 2e; NPV) and specificity (Fig. 2e; SPE). These performance measures assess the diagnostic ability of the models (AUC) as well as the sensitivity and specificity of the models taking into account the population prevalence of the disease (PPV and NPV). For predictive performance comparison, we also built the ACOG criteria-based model based on risk factors constructed from patient characteristics and medical history recommended by ACOG^14^, and computed its AUC using risk score (see Methods).

As the density of data increased across the antepartum period, the median AUC score increased from 0.69 (interquartile (first quartile-third quartile) [IQ]: 0.68–0.70) at week 4 where most of clinical attributes obtained from the patient’s historical information, to 0.92 (IQ: 0.89–0.92) at week 37, which captures nearly all feature values through the pregnancy course. We calculated a median AUC score of 0.82 (IQ: 0.82–0.83) for intrapartum and 0.89 (IQ: 0.89–0.90) for postpartum in the cross-subject validation analysis. In comparison, the ACOG criteria-based model for antepartum achieved a median AUC score of 0.62 (IQ: 0.62–0.63) with high-risk factors and 0.67 (IQ: 0.67–0.68) using all risk factors. We also compared our model PPVs to existing PE risk assessments used as part of standard of care (i.e., population prevalence during the same gestational week) using our models. For example, the PPV for our model at week 4 was 0.04 (IQ: 0.03–0.04) compared to a prevalence of 0.02 (a greater than 2-fold increase). Similarly, the PPV for our model at week 37 was 0.094 (IQ: 0.089–0.104) compared to a prevalence of 0.015 (a greater than 8-fold increase). Complete performance summaries across all models are provided at Table 20 and Supplementary Fig. 4.

### Refining key features during the pregnancy journey

**Fig. 3:**
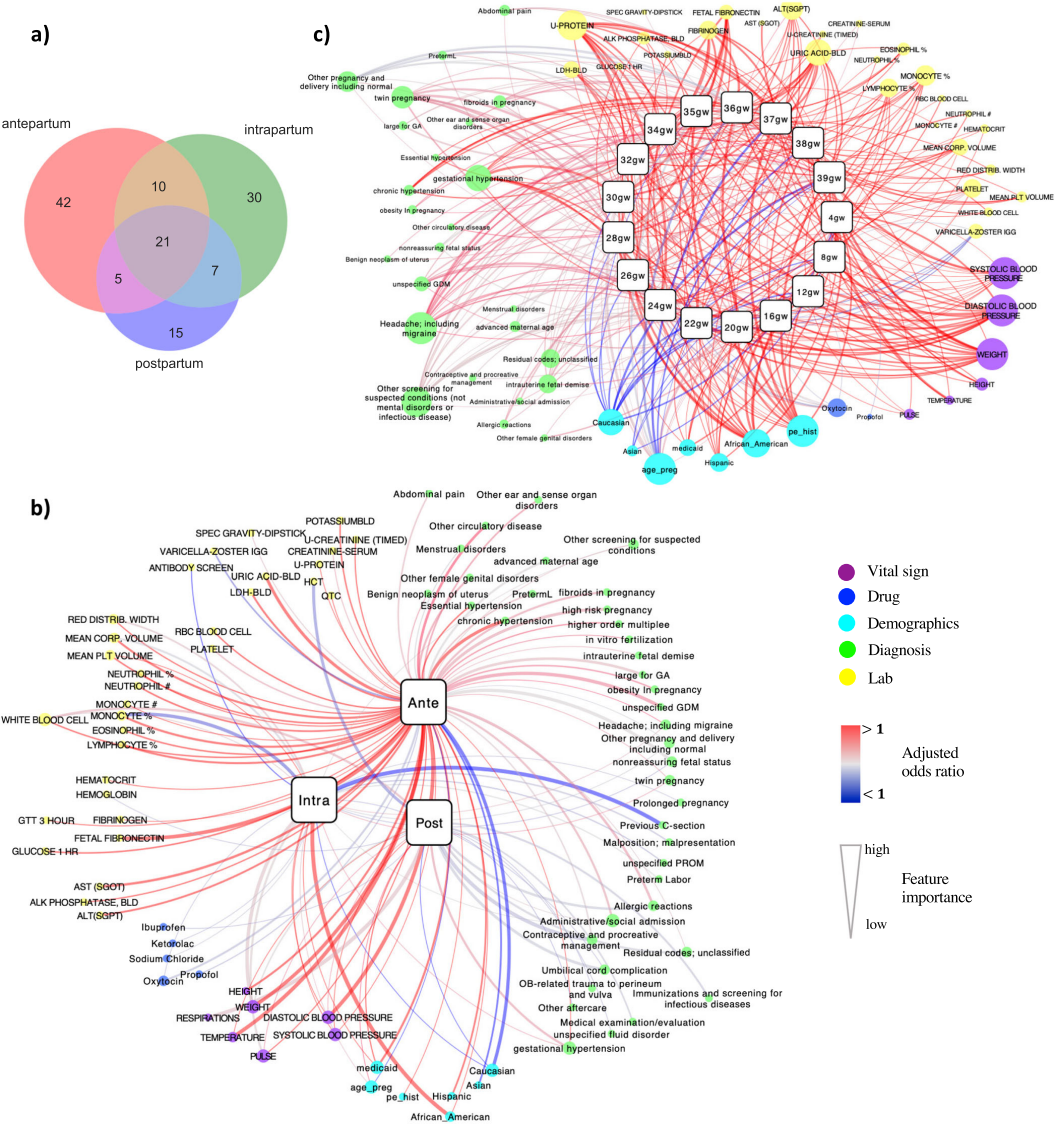
Networks of feature associations through pregnancy. **a** Venn diagram to show common features shared with three pregnancy periods, and specific features to each period. **b** The network to display the associations of selected clinical features with each pregnancy period. **c** The network for the 17 time points in the antepartum. The two networks were constructed by connecting predictive features with respective PE time point. The squares signify different time points of PE, and the round nodes represent the identified predictive features with their sizes proportional to the feature importance. The red edges indicate risk associations (adjusted OR > 1) while the blue edges indicate protective associations (adjusted OR < 1). The edge width reflects the significance of predictive features. Different feature categories are represented with different colors and also laid out together. The networks were visualized using Cytoscape 3.7.2.

We identified 78, 68, and 48 uniquely influential clinical features across the entire antepartum, intrapartum, and postpartum periods, respectively (Fig. 3a). Twenty-one features were significant predictors in all three periods, and 42, 30, and 15 features, respectively, were specific to antepartum, intrapartum, and postpartum. Among the 21 common features, which were enriched for patient demographics and baseline characteristics, we identified 48% features supported in the literature as associating with PE risk, including systolic blood pressure (SBP)^14,18^, diastolic blood pressure (DBP)^19^, weight^18^, maternal age^20^, hemoglobin^21^, white blood cell count^22^, gestational hypertension^14^, PE history^23^, chronic hypertension^22^ and headache (including migraine)^24^ (Supplementary Tables 1–19). Features specific to antepartum were enriched with CBC findings that suggest inflammation and/or infection, such as elevated neutrophil, monocyte, eosinophil, and lymphocyte levels. Intrapartum-specific factors included pregnancy complications such as malposition, malpresentation, premature rupture of membranes (PROM), and sodium chloride (salt) use. Predictors in the postpartum period included many indications relating to follow-up care, such as immunizations, screening for infectious diseases, OB-related trauma, and ibuprofen usage (Fig. 3b).

To better visualize the contributions of the most predictive features across the pregnancy time periods, we further reduced the number of features during intrapartum and postpartum periods to 30 and 24 unique features, respectively, while maintaining the same level of performance (see Methods section). Associations between each clinical feature and PE were visualized by each time period (Fig. 3b), confirming known relationships such as Caucasian and Asian patients being less likely to develop PE, while African American and Hispanic patients were more prone to PE, especially during the intrapartum period (OR:1.25 [95% CI, 1.09–1.43]). Additionally, we identified that patients covered by Medicaid insurance were more likely to develop PE^25,26^. Additionally, we have found features that have not been reported before, such as our identification of pulse rate as a risk factor that was consistently associated with PE in each time period.

To further characterize features we identified as predictive for PE risk, we constructed an interaction network of predictive clinical features and PE across the 17 time points within the antepartum period (Fig. 3c). From the resulting network, we identified clusters of unique lab test features (*N* = 33), diagnoses (*N* = 28), vital signs (*N* = 8), demographics (*N* = 7), and drug prescriptions (*N* = 2). We confirmed well-known risk factors for antepartum PE^14^ (Supplementary Tables 1–17). Moreover, we identified PE biomarkers previously reported in the literature, including fibrinogen^27^, mean platelet volume (MPV)^28^, mean corpuscular volume (MCV)^29^, red cell distribution width^29^, fetal fibronectin^30^, and lactate dehydrogenase (LDH)^31^. Finally, we identified potential novel features that have not been previously reported as associated with PE. For example, median value of varicella zoster virus antibody (IgG) titer is lower in PE patients compared to non-PE patients from 12 to 28 gestational weeks of pregnancy.

### Assessing the dynamic progression of PE associated risk features

**Fig. 4:**
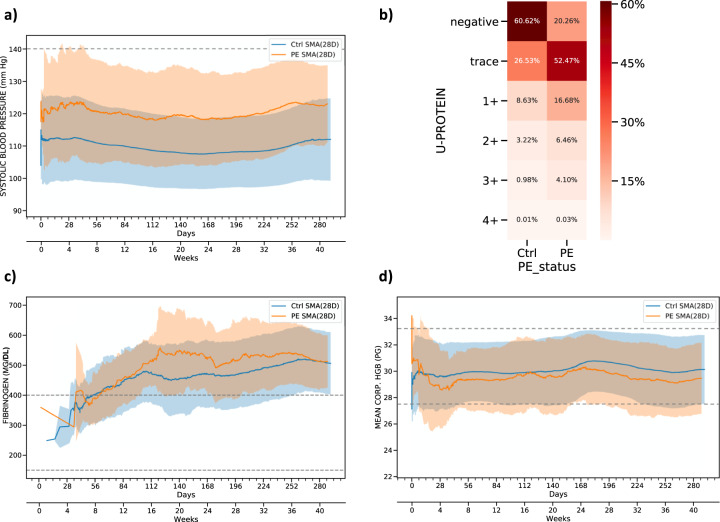
Feature inspection for antepartum based on moving average. **a** 28 days moving average of systolic blood pressure for PE and control patients. The dashed line shows the normal range of systolic blood pressure. **b** Distribution of urine protein for PE and control patients. **c** 28 days moving average of fibrinogen for PE and control patients. The dashed line represents the reference ranges for fibrinogen. **d** 28 days moving average of mean corpuscular hemoglobin (HGB) for PE and control patients. The dashed line represents the normal range of mean corpuscular hemoglobin. In the moving average plots, the shaded areas indicate the standard deviation and solid lines represent the average value across the pregnancy.

To better characterize the dynamic progression of PE features, we generated moving average plots for the significant risk factors, revealing interesting patterns of association even among well-known risk factors. For example, while abnormally high SBP is a well-known risk factor used as a diagnostic marker for PE^18^, by examining longitudinal SBP measures across >100,000 pregnancy journeys, the data show that patients who developed PE in the antepartum period generally had elevated SBP measurements compared to patients without PE, even though the elevated measures fall within a normal range and would not be classified as abnormal during a clinical office visit (Fig. 4a). The average SBP for PE patients in the antepartum time period was only ~120 mmHg^32^, but then consistently through the antepartum period 10 mmHg (one standard deviation of mean from control) higher compared to the control cohort, an important predictive signal for PE picked in nearly all of the models. DBP showed a similar pattern, albeit at a reduced signal strength compared to SBP^18^. Similarly, while protein in urine (U-Protein) is also a well-established diagnostic marker for PE, our data show that the presence of protein in urine even in trace amounts, is a significant predictor for antepartum PE (Fig. 4b). As with SBP, the trace urinary protein levels were supported by our models as a significant predictive feature of PE, even though on their own, recorded at a single visit rather than a longitudinal pattern, trace levels would not be deemed as relevant in current clinical practice.

In addition to the physiologic and urinary findings, our antepartum models also identified and quantified several biomarkers scored in routine laboratory tests, including fibrinogen, blood uric acid, and mean platelet volume (Fig. 3c). Each of these biomarkers exhibited increasing effect sizes in PE cases compared to controls, as measured by adjusted odds ratio over the course of pregnancy journey. These results suggest the corresponding clinical factors predict a greater risk of antepartum PE onset during the later periods of pregnancy. As an example, fibrinogen has been previously associated with PE (especially early onset)^33^. By examining the moving average of fibrinogen along the course of the antepartum time period, we found that the levels of fibrinogen exhibited a moderate increase at 16 weeks in patients who later developed PE (Fig. 4c), suggesting that fibrinogen could be closely monitored over time to enhance the prediction of PE. Along with enhanced utility of known PE risk factors by examining signals longitudinally, mean corpuscular hemoglobin (HGB) was found to be a novel predictor of PE, with slightly lower values observed throughout the antepartum time period in patients who later developed PE (Fig. 4d). Taken together, our PE prediction models were able to recover known and novel clinical factors that enhanced power to predict PE.

### Intrapartum features prioritized by importance based on SHAP values

**Fig. 5:**
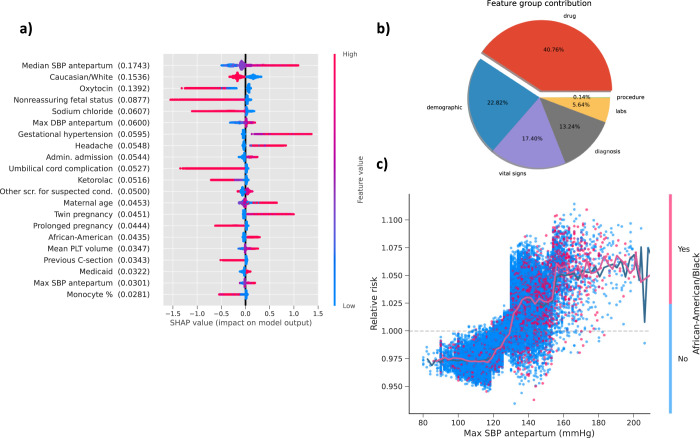
Feature inspection for intrapartum based on SHAP value. **a** SHAP summary plot for top 20 clinical features for PE prediction shows the SHAP values for the most important features from gradient boosting model in the training data. Features in the summary plot (*Y*-axis) are ordered by the mean absolute SHAP values (in the parenthesis after each feature name), which represents the importance of the feature in driving the intrapartum PE prediction. Values of the feature for each patient are colored by their relative value with red indicating high value and blue indicating low value. Positive SHAP values indicate increased risks for intrapartum PE and negative values indicate protective effects to intrapartum PE. **b** The average feature group contribution calculated from averaging mean absolute SHAP values for each feature set. **c** The dependence plot with maximum SBP measured in antepartum versus PE relative risk, along with the interaction of African American race. The plot shows how different values of the feature can affect relative risks and ultimately impact classifier decision. Data points are colored by the African American race. The solid line represents the mean of SHAP values.

We utilized the framework, SHAP values^34,35^, to prioritize the feature contributions to PE predictions by averaging feature importance estimates (Fig. 5a). Median SBP measured in antepartum was the most predictive feature for PE in the intrapartum period followed by Caucasian race and oxytocin administration. We also calculated the average contribution of each clinical category for PE predictions (Fig. 5b). We found medications provided 40.76% predictive power, demographics 22.82%, vital sign contributing 17.40% followed by diagnoses (13.24%), labs (5.64%) and procedures (0.14%). To uncover the relationship between PE risk and changes within a specific feature range, we explored dependence plots which show relative risk (RR) against feature values. To illustrate this point, we provided a representative selection (Fig. 5c), demonstrating PE relative risk in terms of antepartum maximum SBP values and the interaction with African American race. We observed that maximum SBP antepartum tended to become a risk factor after 130 mmHg, and the relative risk values changed rapidly around 130 mmHg.

### Postpartum features reveal novel medication effects related to racial disparities

**Fig. 6:**
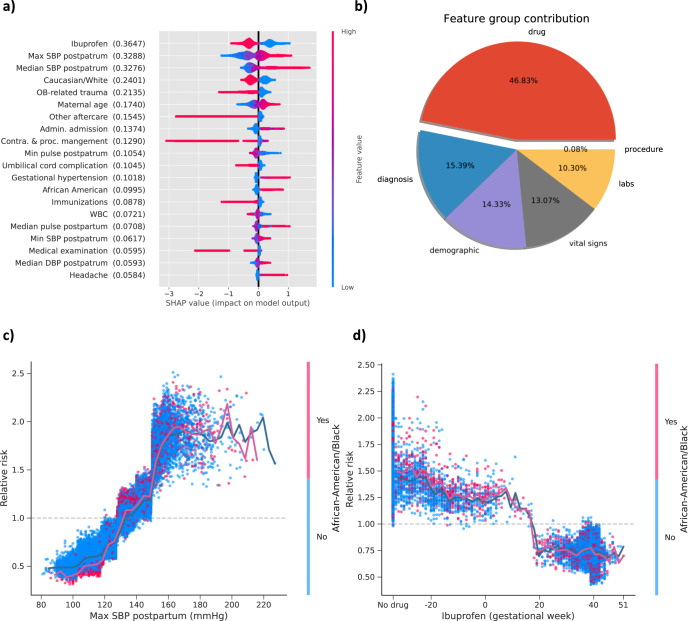
Feature inspection for postpartum based on SHAP value. **a** SHAP summary plot for top 20 features. Features in the summary plot (*Y*-axis) are ordered by the mean absolute SHAP values (in the parenthesis after each feature name), representing the importance of the feature in driving the postpartum PE prediction. Values of the feature for each patient are colored by their relative value with red signifying high value and blue presenting low value. Positive SHAP values indicate increased risks for postpartum PE and negative values indicate protective effects to postpartum PE. **b** The average feature category contribution. **c** The dependence plot of PE relative risk in terms of maximum SBP measured in postpartum. **d** The dependence plot of PE relative risk versus ibuprofen. The SHAP dependence plots indicate how different values of the features can affect relative risks and ultimately impact classifier decision for SBP and ibuprofen stratified by African American race. The solid line shows the mean of SHAP values.

In postpartum period, ibuprofen was the best predictor for PE risk, followed by maximum and median SBP measured during postpartum (Fig. 6a). Both Caucasian race and OB-related trauma showed protective benefit for PE risk reduction. OB-related trauma is common among vaginal deliveries, so this feature likely reflects the protective effect of a vaginal delivery relative to a Cesarean delivery. As a category, medications provided the highest average predictive contribution (46.83%), followed by diagnoses (15.39%), demographics (14.33%), lab tests (10.30%), and procedures (0.08%) (Fig. 6b).

Among predictive features during the postpartum period, we observed that maximum SBP measured in postpartum had a clear effect on the risk of PE (Fig. 6c). The risk of PE increased almost linearly as the elevation of SBP until around 150 mmHg where relative risk steeply increased. Evidently, maximum SBP postpartum would become a risk factor when it exceeded 130 mmHg. Among the patients with maximum SBP ranging from 130 mmHg to 150 mmHg, African American patients were at higher odds to develop PE compared to other races. Among the 18,214 pregnancy journeys in this range, 2978 were African American patients. Within African American race group, the ratio of patients with PE risk (RR ≥ 1) to those without PE risk (RR < 1) was 12.23 while the ratio within other race groups was 3.63. Interestingly, the protective effect of ibuprofen appeared limited to this time period and may increase risk when used prior to pregnancy (Fig. 6d).

### PE predictive model validated in two independent datasets at Mount Sinai Health System

**Fig. 7:**
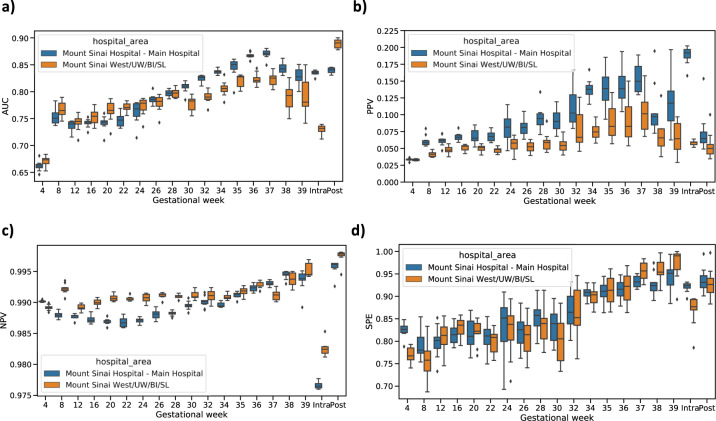
Model validation on two independent datasets in MSHS. **a** Area under receiver operating characteristic curve (AUC) score for each time point. **b** Positive predictive value (PPV), along with preeclampsia risk in the population, at each time point. **c** Negative predictive value (NPV) at each time point. **d** Specificity (SPE) at each time point. Blue boxes indicate the validation in MSH testing set and yellow boxes represent the validation in MSW dataset. The median, interquartile (1st and 3rd) range, and minimum and maximum values, are depicted by the center line, the bounds of the boxes, and the whiskers respectively.

We tested the external validity of our predictive models using two independent datasets, a withheld test set from Mount Sinai Hospital (MSH) and all data collected from Mount Sinai West (MSW). Demographic and clinical characteristics were reported in Table 1 and PE prevalence for each period is listed in Supplementary Table 21. We evaluated performance in these two datasets using four predictive performance metrics, AUC, PPV (positive predictive value), NPV (negative predictive value), and specificity (SPE) (Fig. 7). Other detailed metrics are reported in Supplementary Tables 22–23.

For the MSH test set, we achieved an AUC of 0.66 (IQ: 0.65–0.67) at week 4, which rose continuously as more clinical information became available and reached 0.87 (IQ: 0.86–0.87) at week 37. Consistent with this trend, our intrapartum and postpartum models had AUC scores of 0.83 (IQ: 0.83–0.84) and 0.84 (IQ: 0.84–0.85), respectively. In comparison, we also assessed prediction performance from ACOG criteria-based model for antepartum, AUC score was 0.58 (IQ: 0.58–0.59) using high-risk factors, and AUC score was 0.66 (IQ: 0.65–0.67) using all risk factors. All our models had much higher precision than case prevalence. The lowest PPV we reported was at our first time point, week 4, where a PPV of 0.033 (IQ: 0.032–0.034) was observed and case prevalence was 0.013. By week 37, we observed a PPV of 0.16 (IQ: 0.12–0.15) and case prevalence was 0.007. For intrapartum (prevalence = 0.035) and postpartum (prevalence = 0.008), the PPV was 0.19 (IQ: 0.18–0.20) and 0.08 (IQ: 0.07–0.08), respectively. The median NPV scores for all the periods were at or above 0.98 (Supplementary Table 22). The SPE within antepartum was 0.82 (IQ: 0.81–0.83) at week 4 and boosted to 0.92 (IQ: 0.91–0.93) at week 37. We estimated SPE was 0.92 (IQ: 0.92–0.93) for intrapartum and 0.95 (IQ: 0.94–0.95) for postpartum.

Performance was similar in the MSW test set. AUC score was 0.68 (IQ: 0.66–0.68) at week 4 and increased to 0.82 (IQ: 0.82–0.83) at week 37, compared to 0.58 (IQ: 0.56–0.59) by high-risk factors, and 0.64 (IQ: 0.62–0.66) with all risk factors by the ACOG criteria-based model. Intrapartum and postpartum had AUC scores of 0.74 (IQ: 0.73–0.74) and 0.90 (IQ: 0.88–0.90). PPVs ranged from 0.034 (IQ: 0.033–0.035) at week 4 to 0.086 (IQ: 0.083–0.091) at week 37 compared to existing PE risk (0.016 at week 4 and 0.011 at week 37). All NPV scores surpassed 0.98 on median for every model throughout pregnancy. More details can be found in Table 23 and Supplementary Fig. 5.

### Comparison with published studies

Several previous studies have been developed for the early prediction of PE using either a logistic regression model or a competing risk model by incorporating maternal characteristics, medical history, biochemical markers, and Doppler ultrasound imaging^36–42^. However, these models only included predictors collected before a certain gestational week (ranging from 11 to 24 weeks across various studies), without taking into account that the predictors (especially vitals and laboratory results) might fluctuate across the pregnancy journey. Our proposed predictive models, on the other hand, could assess PE risk at each protocol visit through incorporating the dynamic changes of clinical manifestations during the pregnancy. In addition, some of the risk factors used in the existing models, such as biochemical markers, could not be accessed routinely during the pregnancy visits. To better compare our model with other existing models, we listed the prediction performance from the training dataset of the seven existing models as reported in the manuscripts, along with when the predictors were collected and how the performance was evaluated (Supplementary Table 24). In addition, we also listed the performance of the ACOG model with all risk factors evaluated using MSH training data and estimated the performance of our model at gestational week 16 to match other existing models, and at gestational week 34 to predict PE later on in the pregnancy (Supplementary Table 24). Our model achieved AUC score 0.75 (IQ: 0.74–0.76) and 0.85 (IQ: 0.84–0.91) for gestational weeks 16 and 34, respectively.

Moreover, since some of the known risk factors were not selected by our algorithms, we also performed sensitivity analyses using features from the ACOG criteria in addition to our selected features and to assess potential gains in performance compared to our models as well as ACOG model. The AUC of the models (ACOG and our selected features) had no significant difference from our models (*t*-test *P* = 0.99, *P* = 0.84, and *P* = 0.77 for MSH training set, MSH and MSW test sets, respectively) (Supplementary Fig. 1). Further, we performed analyses using well-established features (preexisting hypertension, history of preeclampsia in previous pregnancy, maternal age, number of babies in the current pregnancy, race/ethnicity, diabetes type I/II, autoimmune disorders, and height) in addition to our selected features and assessed the performance gain compared to our models as well as the model using the well-established features alone. There was no significant difference between our models and the models containing well-established risk factors, with and without and our selected features (*t*-test *P* = 0.98, *P* = 0.72, and *P* = 0.35 for MSH training set, MSH and MSW test sets, respectively) (Supplementary Fig. 2).

## Discussion

This study represents the first data-driven effort to predict PE events across the entire pregnancy journey (antepartum, intrapartum, and postpartum) by comprehensively integrating all clinical characteristics extracted from large-scale EMR data. Our predictive models can identify PE at different time points in accordance with the OB visit protocol, significantly outperforming the ACOG criteria-based model with commonly assessed risk factors. We have tested our developed framework in an independent dataset from a different geographic region and observed comparable performance, demonstrating portability of our PE predictive system to other new facilities.

We captured important features contributing to the PE prediction across pregnancy time points. To provide maximum interpretability to physicians, we calculated moving averages across time points for key features, as well as SHAP values to indicate relative importance of individual features to overall risk prediction. We identified features common across all three pregnancy periods and features unique to each period. Specifically, other than common features, CBC-related characteristics dominated in antepartum; pregnancy complications associated with intrapartum; follow-up care impacted postpartum.

Some of the findings give further credence to the underlying mechanisms associated with preeclampsia, especially that of dysregulated inflammatory processes^43^. We found several laboratory markers that are complementary predictive factors of preeclampsia in our model and are routine available in the antenatal period. Elevated neutrophil, monocyte, eosinophil, and lymphocyte levels were noted in the antepartum time frame. Fibrinogen, aside from being an important molecule in coagulation, also has an important role in inflammation and serves as an acute-phase protein^44^. Additionally, the temporal relationship of these markers allows for a more specific and nuanced prediction of preeclampsia at multiple time points in pregnancy. Previous prediction models have focused on risk factors at a single time point in pregnancy^37,38,40,42,45^. In contrast, our model incorporates risk factors over time and allows for refined prediction as the pregnancy journey progresses and postpartum. For illustration, we compared our model at gestational week 16 to other models in the literature, as well as our model at gestational week 34 (Supplementary Table 24). The performance of our model at gestational week 16 was very similar to most of the predictive models for early screening of PE, which included variables that could be obtained during routine visits, such as maternal characteristics, medical history, and laboratory results^37,38,40,42^. While Odibo et al. performed comparably to our week 16 model, in addition to the variables from routine visits, their model also included Doppler ultrasound and biochemical markers^39^. Our model at gestational week 34 had the best performance among all the models listed. Although the competing risk model from Wright et al. and the logistic regression from Yu et al. performed similarly, these models also required Doppler ultrasound result and/or biochemical markers, which is usually challenging to access using EMR data from routine pregnancy visits^36,41^.

Of interest, ibuprofen was noted to show protective association in the postpartum preeclampsia model, which further the findings from a double-masked randomized trial that ibuprofen did not lengthen the duration of severe-range hypertension in women with PE with severe features^46,47^.

Other interesting findings included several insights regarding blood pressure measurements. One advantage to the algorithm is that it does not require any special BP measurements, beyond those commonly performed in the office and recorded in the medical record. Rather than the specific BP measurement, it is the trajectory that consequent signal that drives the algorithm. SBP was a more powerful driver vs DBP, which has been noted previously^48^. However, in our study, we were able to confirm the importance of SPB > 130 mmHg as an important threshold for concern. This finding was readily apparent and lends further support to the those who consider the 140/90 threshold to be too high^48^, especially in light of the new AHA recommendations^49^. The association with elevated SBPs and African American race, particularly in the postpartum period, also affirms recent literature that suggests a different BP pattern in African American women following delivery, which warrants further research and assessment. Medicaid has been picked as a significant feature to PE, indicating that these patients may be at increased risk because of limited access to healthcare or other barriers due to low socioeconomic status.

While features associated with inflammatory processes and BP were anticipated, there were other features that could be potentially novel and merits further investigation. The median value of varicella zoster virus antibody (IgG) titer was significantly lower in PE patients compared to non-PE patients from 12 to 28 gestational weeks of pregnancy. This association suggests that higher IgG against varicella zoster, developed from vaccination prior to their pregnancy, or an underlying mechanism, may indicate protective association with PE^50^.

Some of the risk factors from ACOG guideline, such as diabetes, systemic lupus and other autoimmune disorders were not selected for our models, which might be due to (1) ICD diagnosis code system is not perfect to identify patients with the conditions; however, this approach has limitations in capturing all the patients of interest (more details below); (2) we did not consider the severity of the conditions in the model. That is, from our data, we would only know that a patient had been diagnosed with diabetes, but we cannot know whether this patient is a relatively ill or healthy patient (e.g. through different adherence to medications and dietary recommendations).

Our study had several limitations. As our clinical data was extracted from MSH, which is close to other medical centers in the area, patients may have received prenatal care at other nearby hospitals or clinics but then chose to deliver at MSH, resulting in the loss of valuable information from our EMR system. Moreover, patients might not come for follow-up care after discharge. To tackle this issue, we designed sparsity filters to ignore some journeys only with minimal available features, e.g. journeys only with demographics. Nonetheless, even patients receiving care at a single facility will often have missing values. Here, since the gradient boosted tree algorithm can accommodate the missing values, we chose not to explicitly impute them, as this better reflects clinical practice where some patient information might be not available. Additionally, our methodology used ICD9/10 codes to identify maternal comorbidities and excluded detailed physician notes. As a result, we may have excluded some “over the counter” medications, comorbidities, and/or diagnoses. This is likely due to the intended function of ICD codes being for billing, not diagnostic purposes^51^. Specifically, previous studies have shown that using ICD codes alone to identify preeclampsia patients performed poorly when compared to the gold standard of chart review^52,53^. Indeed, to identify as many PE cases as possible, ICD codes should be combined with EMR records (such as vitals and laboratory results). Being aware of these limitations, we took the more comprehensive digital phenotyping approach to better capture PE cases.

Extensive research has identified three important biomarkers for preeclampsia^54–56^, mean arterial pressure (MAP), uterine artery pulsatility index (UtA-PI), and serum placental growth factor (PlGF). Due to data access restrictions on identifiable data such as ultrasounds, our PE prediction system was developed solely based on structured EMR data. However, we achieved similar or even better prediction performance compared to the models incorporating these biomarkers^57^. Our methodology allows for the incorporation of biomarkers into our current PE prediction system that would be expected to generate more robust performance. Similarly, certain ‘Omic’ data has shown promise for identifying PE^4–9^, but this type of data is not collected routinely in a clinical setting. Although further studies are needed to incorporate known biomarkers as well as ‘omics’ data, some of which are still investigational while others such as PlGF are already in clinical use, our algorithm—based on EHR data alone—has significant potential implications for clinical care and management.

Our results showed both common features shared among all periods, and unique features specific to each pregnancy period exist, suggesting significant pathophysiologic differences in each pregnancy period in terms of risk for PE. We have confirmed previously known risks to PE, and also uncovered potentially novel connections between clinical features and PE, some of which are supported by other clinical and experimental data. Furthermore, we have validated our models with similar predictive performance on two independent test datasets with population diversity. The results open the door for optimizing monitoring tools to mitigate risks and for individualizing assessment based on patient risk profiles. In addition, this paper provides the most complete assessment of vital sign patterns and trajectories in patients with and without preeclampsia. We have demonstrated that by harnessing the power of data science, we can enhance predictive PE algorithms throughout the pregnancy journey. Hopefully, with continued research, better screening performance based on precision monitoring strategies, will ultimately lead to preemptive clinical strategies and improved perinatal outcomes.

## Methods

The aim of the study was to develop and validate a prediction tool to screen for and monitor patients at risk for PE using clinical information from 108,557 pregnancies at MSHS in New York City, a large health system with a highly diverse population. We built and implemented a digital phenotype for PE based on ACOG recommendations^14^ to incorporate multiple diagnostic tests and criteria. We performed data processing, model training and validation, and results interpretation for predicting PE risk and interpreting associations between clinical features and PE.

### Data source and pregnancy journey construction

We utilized de-identified EMR from MSHS. By March 2019, the system contains records for >9 million unique patients since 2002. The Mount Sinai EMR covers heterogeneous clinical information including patient characteristics, diagnosis, procedure, medications, vital signs, and lab tests for visits. We selected patients from Mount Sinai Hospital (MSH) and Mount Sinai West (MSW, we additionally added Mount Sinai Upper West, Mount Sinai St. Luke and Mount Sinai Beth Israel together) who are biologically female between age 12 and 50 with either: (A) diagnosed with labor and delivery related International Classification of Disease 9th or 10th billing codes; (B) has vaginal or cesarean section delivery Current Procedural Terminology 4th billing codes; or (C) admission records to labor and delivery facility. We identified 114,757 standalone delivery events for 88,907 unique patients, with 1.29 deliveries per patient. We extracted gestational week mentioned in the admission records to labor and delivery facility, admit reason for inpatient and outpatient visits, and ICD9/10 diagnosis codes associated with specific gestational weeks. Then, we calculated the pregnancy date as gestational week report date—7 * gestational week. We were able to find gestational week records and calculated the accurate pregnancy dates for 114,312 deliveries (83,954 unique patients) (Fig. 1a), with the average age of 31.06 (std: 6.09) at pregnancy.

We extracted patient demographics, diagnoses, prescription drugs, anesthesia-involved procedures, vital signs, and lab tests from MSDW EMR for patients in the study cohort (Supplementary Table 24). For each journey, we collected data from as early as 8 months before the pregnancy to as late as 10 weeks past the delivery. This (A) minimizes the influence of clinical signals associated with previous delivery yet preserves as much as possible prior-pregnancy information of the patient; and (B) corresponds to the timeline of preeclampsia development, which can happen as late as 10 weeks postpartum.

The demographic information includes age at the pregnancy, race, tobacco usage, alcohol usage, recent preeclampsia history, and Medicaid insurance. For patient who had reported multiple race groups, we assigned them to all race groups they had reported. We considered the patient under tobacco or alcohol usage, if they had reported such use during or before the 10 weeks after delivery.

The original diagnosis records in the MSHS EMR contains 14,688 ICD9/10 codes for the Pregnancy-Delivery (PD) journey cohort. We grouped these ICD9/10 codes into 279 (of 285) Clinical Classification Software (CCS) single-level categories^58^ and 121 reproductive disease categories defined by our OB/GYN. This helps to reduce dimensionality of heterogeneous diagnosis features to the granularity level suitable for building machine models and interpret clinical meaning.

We did not differentiate prescriptions of the same drug with difference dosage or under different brand names, and common ingredients of the different drugs may impact development of preeclampsia in the same way. Therefore, we mapped 8682 unique prescribed drug names to 1618 drug ingredients concepts registered in the RxNorm, using the RxNav API from National Library of Medicine [https://rxnav.nlm.nih.gov/APIsOverview.html].

The PD journey cohort contains 718 unique CPT codes for anesthesia-involved procedures, which were directly retrieved from the EMR.

We collected vital signs including pulse, systolic blood pressure, diastolic blood pressure, temperature, respirations, weight, height, O_2_ saturation, and pain scores for each journey and unified unit of measurements to Beats/Min, mmHg, Fahrenheit degree, kilogram, centimeter, percentage, and 10-point scale respectively. For all but pain scores, we removed vital values beyond the range of Guinness World Records.

Patients in the PD journey cohort took a total of 603 lab tests. We normalized the lab names by mapping free text 603 lab names to 348 LOINC codes, using the RELMA software [https://loinc.org/relma/] and manually validated the mapping results. We unified the unit of measurements for the same tests to the default unit of the corresponding LOINC. Out of all labs, 514 (283 LOINC) has numeric values, for which we unified the unit of measurements. For the 89 lab tests (65 LOINCs) that has descriptive text values, we unified nominal values and encoded nominal values to ordinal numbers based on test strip description^59^ and color charts (e.g., “negative” -> 1, “trace” -> 2, “small” -> 3, “moderate” -> 4, “large” -> 5).

This data usage is approved by institutional review board (IRB) of Icahn School of Medicine at Mount Sinai: IRB-17-01245, who determined that the research does not involve human subjects and granted a waiver of consent based on the nature of the project, including the use of previously collected, de-identified data.

### Digital phenotyping for PE

The World Health Organization recommended patients meeting the following criteria being diagnosed for preeclampsia: (1) Persistent hypertension and (2) Development of substantial proteinuria^60^. In Mount Sinai Hospitals, OB/GYNs used diastolic blood pressure (DBP) of 90 mm hg or systolic blood pressure (SBP) of 140 mm hg as the threshold for hypertension. From ACOG guideline, we added the following clinical features: platelets counts, creatinine, liver function enzymes (AST/ALT), proteinuria, and related diseases such as headache, visual disturbances, pulmonary edema, eclampsia, and seizure (Fig. 1b).

We implemented a diagnosis and rule-based digital phenotyping algorithm to identify PE patients (Fig. 1b). We first identified 2291 patients who were diagnosed with PE ICD9/10 codes between the gestational weeks of 20 and 10 weeks after the delivery and used the first date of the diagnosis as the PE onset date. Additionally, we have implemented additional criteria from the ACOG guidelines to capture the undiagnosed PE cases which were not coded by ICDs (Fig. 1b). We checked if the patients had repeating high blood pressure (≥140/90 mm hg for SBP/DBP and occurred at least twice) within 3 days, and then checked if they had ICD9/10 codes and lab test results indicating the development of proteinuria which happened within 3 days of the first repeating high blood pressure. We identified 6279 patients who met both criteria to be classified as PE. We used the first day of high blood pressure as the PE onset date for these patients. If the patient were not diagnosed with PE and did not meet the hypertension—proteinuria defined criteria within pregnancy and until 10 weeks after the delivery, they were defined as the control group. After examining 2291 patients who were diagnosed with PE ICD9/10 codes, we found 91.36% of them have either repeating high blood pressure above the threshold value or diagnosis and lab results of proteinuria, suggesting the criteria of the vital and laboratory measures from our digital phenotyping are good indicators for PE diagnosis. Including both ICD codes and comprehensive EMR data in digital phenotyping can allow for the identification of more patients than just using diagnosis codes alone.

Based on when the preeclampsia occurred, we further split patients into three sub-types: 1790 patient PE in the antepartum (before admission for labor and delivery), 5315 patients in the intrapartum (between admission of labor and delivery), and 1,020 patients in the postpartum (after delivery).

### Experimental design

To train predictive models for PE along the pregnancy journey, we divided the journey into 19 time points including 17 time points for antepartum (5 monthly visits spanning weeks 4–20, 7 biweekly visits spanning weeks 22–34, and 5 weekly visits spanning weeks 35–39), and intrapartum and postpartum periods as two independent time points with respect to the pathophysiology of PE. We outlined our entire workflow in Fig. 1. We collected the clinical features between 8 months before pregnancy and the time point (the protocol visit) to build a model that could predict PE risk after the time point. We generated the PE ground-truth labels based on our digital phenotyping algorithm which only used the clinical information after the time point. Therefore, there was no information leakage between the training data and the data generating ground-truth labels. We also excluded the PE cases which already happened before each time point when we built the time series model. We listed the PE prevalence, sample size, number of available features, and percentage of missing values for the Mount Sinai Hospital (MSH) training set, the Mount Sinai Hospital (MSH) test set, and the Mount Sinai West (MSW) test set in Supplementary Table 21, respectively.

We split the data collected from MSH into a training set (60%) and a test set (40%) and were sure that the pregnancy journeys of a single patient only belonged to either the training set or the test set to avoid any bias. We did not split the data using stratified sampling, but the percentage of PE cases in both training and testing is similar (as shown in Table 1). We trained our models using the training set for each pregnancy visit (time point) in antepartum and each of pregnancy periods. We first divided the training set into ten folds with respect to patients using “StratifiedGroupKFold” to prepare for cross-subject validation. More specifically, the pregnancy journeys of a patient could only belong to one-fold to avoid the information leakage and to mimic the clinical settings. Considering our imbalanced labels, we also employed stratified sampling to ensure that relative class frequencies were approximately preserved in each training and validation fold. We performed feature engineering, feature selection, hyperparameter tuning and trained the best model using the selected best hyperparameters within each cross-subject validation. More specifically, within one cross-subject validation, we conducted hyperparameter tuning 100 times through another 10-folds cross-subject validation strategy that also included feature engineering and selection to identify the best hyperparameters, and then we used the chosen best hyperparameters to train the final best model using all the training data (i.e. 9 training folds) in this cross-subject validation. We evaluated the trained model performance on the held-out fold in this cross-subject validation. We repeated the above process on the other 9 cross-subject validations. In total, we obtained 10 final best models, each from a cross-subject validation (see Supplementary Fig. 6). We reported the cross-subject validation performances with median and interquartile range (first and third quartile) of the 10 final best models. We then validated our final established models on two independent datasets: the 40% held-out test set from MSH and independent MSW set available until 2019 (including Mount Sinai Beth Israel, Mount Sinai West, Mount Sinai St. Luke and Mount Sinai Upper West) at each time point, and computed the median and the interquartile range of the performance metrics. All population characteristics for each dataset are shown in Table 1. We have reported AUC, SPE, SEN, PPV, and NPV for our model validation performance and comparison with current standard of care, a ACOG criteria-based model (Supplementary Table 20, 22, 23 and Supplementary Figs. 4 and 5).

### Feature engineering

For diagnoses, drug prescriptions and procedures, we selected first timing (gestational week) as the feature value which could provide the timing information to the machine learning models compared with the form of binary feature. In order to distinguish the mode of delivery, we identified the journeys associated with the Cesarean section using both diagnosis and procedure codes, and the vaginal delivery by the corresponding procedure codes, thereby leading to two additional features.

We split the vital sign data into three ranges on par with the definition of the three pregnancy periods that might help capture the explicit contribution of pregnancy period information to the model predictions. In each range, we calculated the maximum pain score for the journeys if applicable, and also included the minimum, median, and maximum values for other numerical vital sign values observed in the interval. We observed that different journeys involved various lengths of available vital sign data, which increased the difficulty of directly injecting these time-related data into the prediction models. To unify the data length and also account for the time-related information, we applied the functional principal component analysis (FPCA) method^61^ to features including diastolic blood pressure, systolic blood pressure, O_2_ saturation, pulse, respirations, temperature, and weight. The FPCA method is able to find the functional principal components and their functional principal component scores representing the variations of time series curves explained by the components which naturally keep distinct information in the time series data. We computed the top 10 functional principal component scores with R package fdapace^62^ as the additional features for the journeys, if available, to interpret the time-related vital sign features.

For lab features, we used the similar process as vital signs that we obtained the maximum ordinal values for journeys and statistical values (minimum, median, and maximum) for other numerical lab features in every range. As the functional principal components are approximated with the summation of basis functions, e.g. B-spline, we chose the lab features at least with >3 data points to perform the FPCA, otherwise, the program would be aborted. Based on this principle, we finally selected 15 lab features and calculated the top 10 functional principal component scores as the additional features for each selected lab feature.

We performed the feature engineering at each time point where we collected data from 8 months before pregnancy up to the specific time point. Collectively, we concatenated all built diagnosis, medications, procedure, vital sign, and lab features along with the demographic. We obtained the number of features ranging from 2989 to 3294 for 17 time points in antepartum, 4136 for intrapartum, and 5391 for postpartum (Supplementary Table 21).

### Feature selection

Since we attained a large volume of features, it is prone to make our final predictive models be overfitted to the training data. Consequently, we performed three feature selection methods: (1) penalized logistic regression with the adaptive LASSO^63^, (2) univariate analysis, and (3) decision tree-based models (XGBoost and random forest)^64^ separately to select a set of important features from each algorithm. Given that we marked diagnosis, drug and procedure features as zeros when patients’ records were not presented in the journeys, we performed the adaptive LASSO on the diagnosis, drug and procedure features to recognize the important features with respect to sparse coefficients and the corresponding *p*-values. Considering the proportion of missing values in vital sign and lab features, we utilized the univariate analysis to obtain the coefficients and *p*-values. Specifically, we combined all the demographic features with a single vital sign or lab feature each time to train a logistic regression model, in which the journeys with missing values were not considered, and the vital sign or lab feature required to 10 or more valid values. For adaptive LASSO and univariate analysis, we picked features with *p*-value < 0.05. We also exploited a random forest model and a XGBoost-trained gradient boosted decision tree model using all the features without the imputation of missing values. For both tree-based models, we used bootstrap sampling with replacement using 80% of the sample, and calculated the feature importance scores. We then picked the features ≥ 75th-percentile from the two tree-based models. Finally, we only selected the intersected features from all the methods (LASSO, univariate analysis, XGBoost, and random forest) to build the predictive models.

### Learning algorithm

In light of the complex nonlinear interactions among the extracted features, we employed gradient boosted tree models^35,65^. These models are able to address the missing values inherently that are ubiquitous in the EMR and also the subsequently retrieved clinical features, such that we could avoid the basis/variance induced by imputing these missing values via the conventional approaches; e.g. mean, median, maximum and minimum, etc. We also utilized LightGBM^66^, a high-performance implementation of gradient boosted tree models, to fit our clinical models and then predict the corresponding targets, specifically, the binary classification in our PE prediction. We used the hyperparameter optimization package Hyperopt^67^, on the basis of Bayesian optimization approaches, to automatically choose the optimal hyperparameters, including learning rate, number of trees, depth of trees, number of leaves, sample rates, L1 and L2 regularization, and number of cases in leaf nodes in the search space with the best performances on the designated metrics.

### Model interpretation and network

Interpretability is critical in clinical settings to explain the specific impact of each feature on the predictive results, not only at the global level (the overall feature impact on the model output) but also at the local level (the influence of each feature on individual sample prediction)^35,65,68^. The Shapley value has been widely used to explain machine learning model outputs in clinical fields and capture the underlying clinical feature attributions and influences on the clinical predictions, such as with chronic kidney disease^62^. Hence, we employed Shapley values realized by SHAP python package to obtain both local and global interpretability. The Shapley values are attributed to game theory, and each feature in the predictive model functions as a player in a coalition. Locally, the Shapley value of each feature value represents the *direction* and the *size* of the contribution for the value towards to the difference between the predicted values and the baseline value, i.e., the expected value of the model when we do not have any information on any features. Globally, Shapley values evaluate the overall contribution of each feature to the model output by averaging across all the samples. In other words, we used Shapley values to quantify the overall contributions of the selected features in our predictive models as well as how the value of the feature contributes to the predicted value in our samples.

Generally, the outputs of the Shapley values using the TreeExplainer from SHAP package are log odds of the predicted values relative to the baseline value, which are additive. To draw the dependence plots, we transformed from the logit space into the probability space with a sigmoid function and calculated the relative risk (*RR*) score since RR is more meaningful and is more broadly used in the clinical fields^69^. In the logit space, the Shapely values can be expressed as

1 $$f\left( x \right) = \phi _0 + \mathop {\sum }\limits_{i = 1}^M \left( {\phi _i} \right)$$

where *f(x)* represents the Shapely value (log odds) of sample *x*; *ϕ*_0_ is the base value representing the population prevalence; *ϕ*_*i*_ is the Shapley values for each feature capturing the difference between the expected model output and the current prediction output; *M* is the number of features. To display the dependence relationship for a single feature, we only computed the relative risk score through the Shapley values of that feature as follows

2 $$RR_i = \frac{{\sigma \left( {\phi _0 + \phi _i} \right)}}{{\sigma \left( {\phi _0} \right)}}$$

where *σ* is the sigmoid function. We could also aggregate certain related features into a higher level to investigate the corresponding overall feature effect on the model output. To this end, we only need to replace *ϕ*_*i*_ with $\mathop {\sum}\nolimits_{i \in S} {\phi _i}$, where *S* is the subset of features desired to be grouped.

Furthermore, we constructed networks connecting predictive features with respective PE, including 17 gestational weeks during antepartum as well as intrapartum and postpartum. It is worth noting that, to better visually illustrate the important features and their associations with each pregnancy period in the network, we reduced the unique feature number from 68 to 30 for intrapartum and from 48 to 24 for postpartum, respectively, by adding features one by one based on the rank of SHAP importance until the prediction performance became flat (Supplementary Fig. 3 show the feature sweeping where the features were derived from the unique features). The nodes in the network represent the stages of PE and identified predictive features. The edges in the network reflect two layers of information: feature importance and adjusted odds ratio. We applied grouped attribute layout in Cytoscape 3.7.2 to draw the network, with node sizes proportional to their degrees, edge width proportional to the feature importance and edge color correspond to adjusted OR. Two networks are visualized: one with different time points across antepartum and one with aggregated antepartum models, together with intrapartum and postpartum models. For simplicity, features that are predictive to only one-time point in antepartum are removed from the visualization.

### Current standard of care: ACOG criteria-based model

To evaluate PE risk in the clinical practice, we assessed the predictive model performance based on high-risk factors and all risk factors (including high and moderate risk factors) recommended by ACOG^14^. We treated each risk factor as a binary feature and calculated a risk score for every pregnancy journey in the corresponding cohort by summing the risk factors. The risk factors are subdivided into high-risk factors and moderate risk factors which are described as follows^14^.

High-risk factors:

- History of preeclampsia, especially when accomplished by an adverse outcome
- Multifetal gestation
- Chronic hypertension
- Type 1 and 2 diabetes
- Renal disease
- Autoimmune disease (i.e. systemic lupus erythematosus, the antiphospholipid syndrome)

Moderate risk factors:

- Nulliparity
- Obesity (body mass index >30)
- Family history of preeclampsia (mother or sister)
- Sociodemographic characteristics (African American race, low socioeconomic status)
- Age 35 years or older
- Personal history factors (e.g., low birth weight or small for gestational age, previous adverse pregnancy outcome, >10-year pregnancy interval)

We establ﻿ished ACOG criteria-based models based on high-risk factor and all the risk factors (high and moderate risk factors), respectively. We obtained the AUC using the risk score and implemented bootstrap sampling (sampling with replacement to sample 90% of the data 1000 times) to evaluate the mean and interquartile range of the AUC values. We had initially included all the risk factors listed on ACOG guidelines for feature selection; however, these risk factors were not selected in our pipeline. To better compare the static ACOG model and our proposed models with dynamic characteristics, we also built a LightGBM model by only using ACOG-related risk factors available at the first protocol visit (week 4) and another model by forcing ACOG-related risk factors to be included in our proposed model. We then evaluated the performance gain of the model including both ACOG-related risk factors and the selected (by pipeline) dynamic features against the proposed models and the ACOG-risk-factor-only LightGBM model.

### Reporting summary

Further information on research design is available in the Nature Research Reporting Summary linked to this article.

## Supplementary information

Supplementary Material

Reporting Summary

## Data Availability

The clinical data here were used under license from Mount Sinai Data Warehouse in the current study. As a result, this dataset is not publicly available. Qualified researchers affiliated with the Mount Sinai Health Systems may apply for access to these data through the Mount Sinai Health Systems Institutional Review Board.

## Code availability

We used several open-source libraries to build our machine learning model, namely ‘fdapace’ (https://github.com/functionaldata/tPACE) in R for FPCA and LightGBM (https://lightgbm.readthedocs.io/en/latest/) and scikit-learn (https://scikit-learn.org/stable/) in Python, and will release the code under the CC BY-NC-SA 3.0 license (https://creativecommons.org/licenses/by-nc-sa/3.0/). However, our data collection, cleaning and quality control framework made use of proprietary data structures and libraries, so we are not releasing or licensing this code. We provided implementation details in the methods section to allow for independent replication.
