# Supplementary material for: Improving preeclampsia risk prediction by modeling pregnancy trajectories from routinely collected electronic medical record data
Source: NPJ Digit Med. 2022 Jun 6;5:68. doi: 10.1038/s41746-022-00612-x (PMC9170686; doi:10.1038/s41746-022-00612-x)
Supplement: Supplementary file 1 — Supplementary Material [file 41746_2022_612_MOESM1_ESM.pdf]

Supplementary Material

Improving Preeclampsia Risk Prediction by Modeling Pregnancy Trajectories from Routinely Collected Electronic Medical Record Data

Shilong Li[1†], Zichen Wang[1†], Luciana A. Vieira[2], Amanda B. Zheutlin[1], Boshu Ru[1], Emilio Schadt[1], Pei Wang[4], Alan B. Copperman[1,2,3], Joanne Stone[2], Susan J. Gross[1,4], Yu-Han Kao[1], Yan Kwan Lau[1], Siobhan M. Dolan[2,4], Eric E. Schadt[1,4*], Li Li[1,4*]

[1]Sema4, Stamford, CT, USA.

[2]Department of Obstetrics, Gynecology, and Reproductive Science, Icahn School of Medicine at Mount Sinai, New York, NY, USA.

[3]Department of Genetics and Genomic Sciences, The Icahn Institute for Genomics and Multiscale Biology, Icahn School of Medicine at Mount Sinai, New York, NY, USA.

[4]Reproductive Endocrinology and Infertility, Reproductive Medicine associates of New York, New York, USA.

[†]These authors contributed equally to this work

[*]Corresponding author. E-mail: li.li@sema4.com; eric.schadt@sema4.com

Supplementary Figures

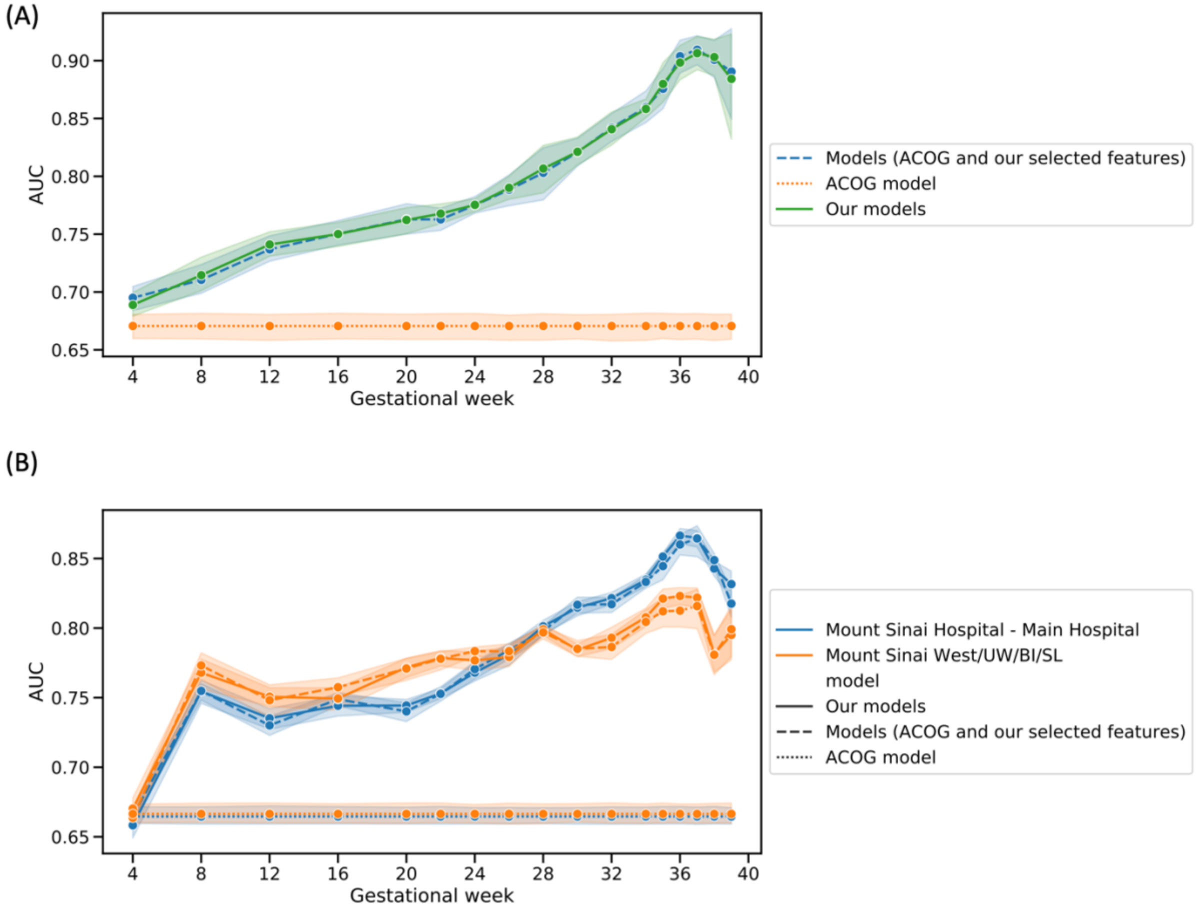

Supplementary Figure 1: Model performance comparisons and gains among our models, models with ACOG criteria on top of our selected features, and the model using ACOG criteria alone. (A) training performance at training dataset; (B) test performances at two independent datasets

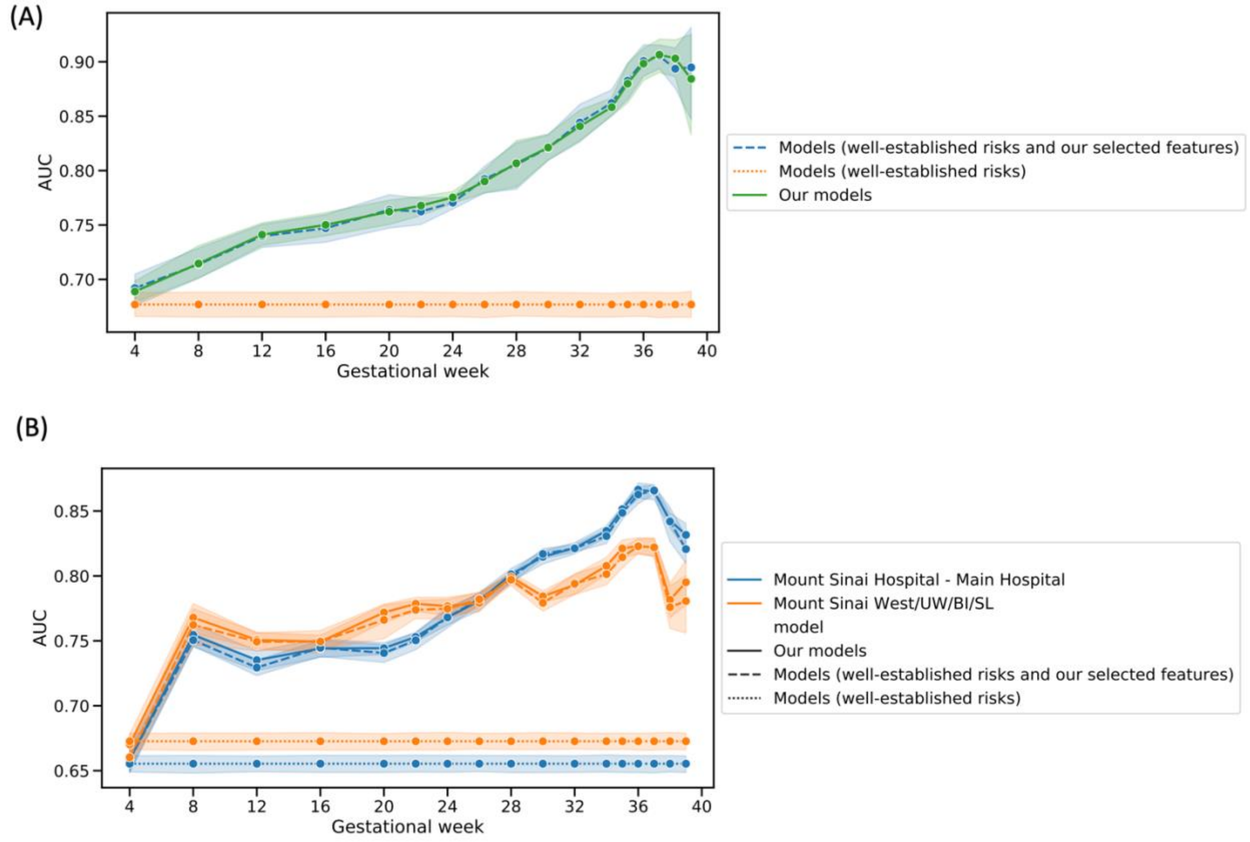

Supplementary Figure 2: Model performance comparisons and gains among our models, models with well-established risks on top of our selected features, and the model using well-established risks alone. (A) training performance at training dataset; (B) test performances at two independent datasets

(A)

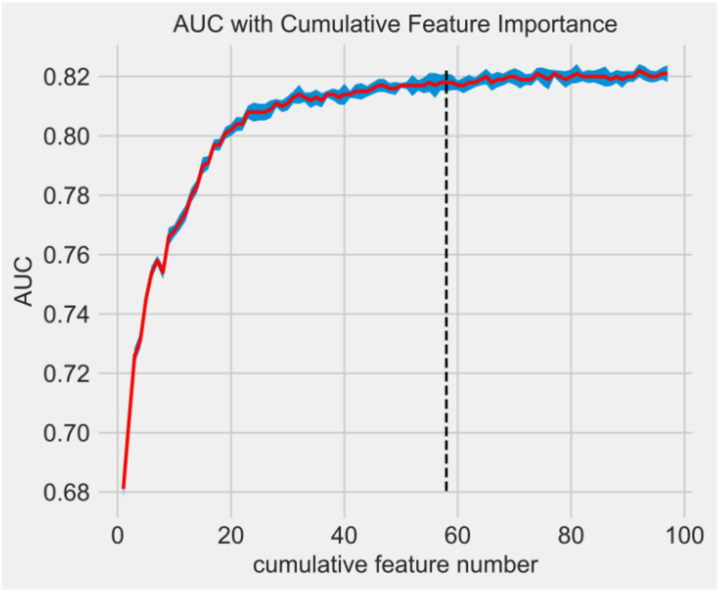

(B)

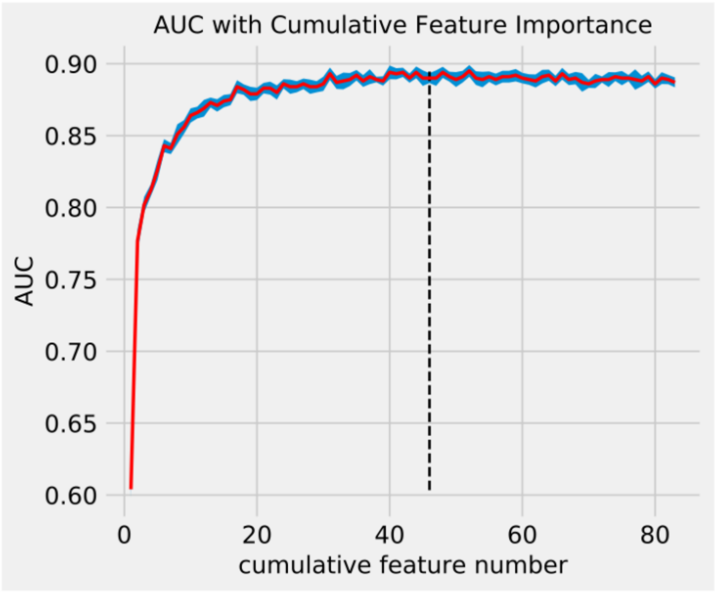

Supplementary Figure 3: AUC score of features cumulation. (A) intrapartum; (B) postpartum

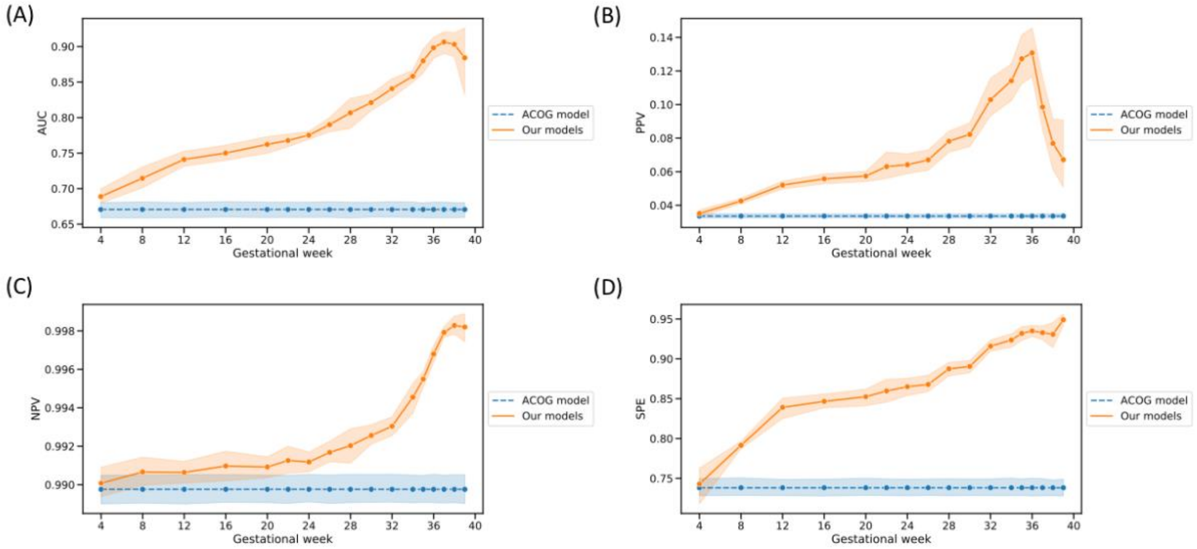

Supplementary Figure 4: Model performance comparisons between our model with ACOG model using the MSH training dataset. (A) Area under receiver operating characteristic curve (AUC). (B) Positive predictive value (PPV). (C) Negative predictive value (NPV). (D) Specificity (SPE)

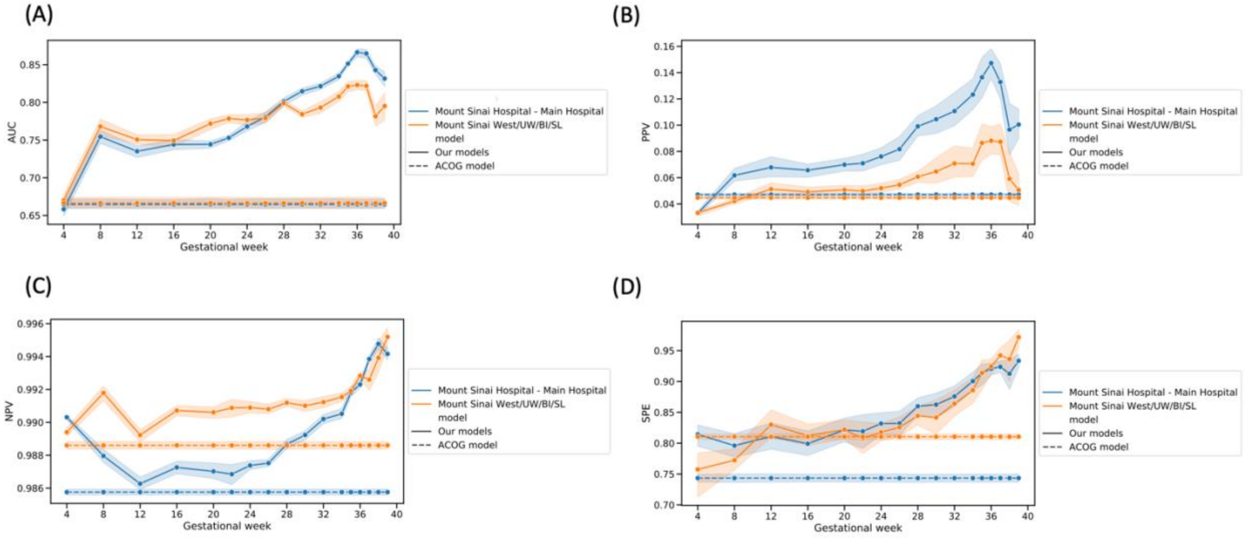

Supplementary Figure 5: Model performance comparisons between our model with ACOG model in the two independent datasets. (A) Area under receiver operating characteristic curve (AUC). (B) Positive predictive value (PPV). (C) Negative predictive value (NPV). (D) Specificity (SPE)

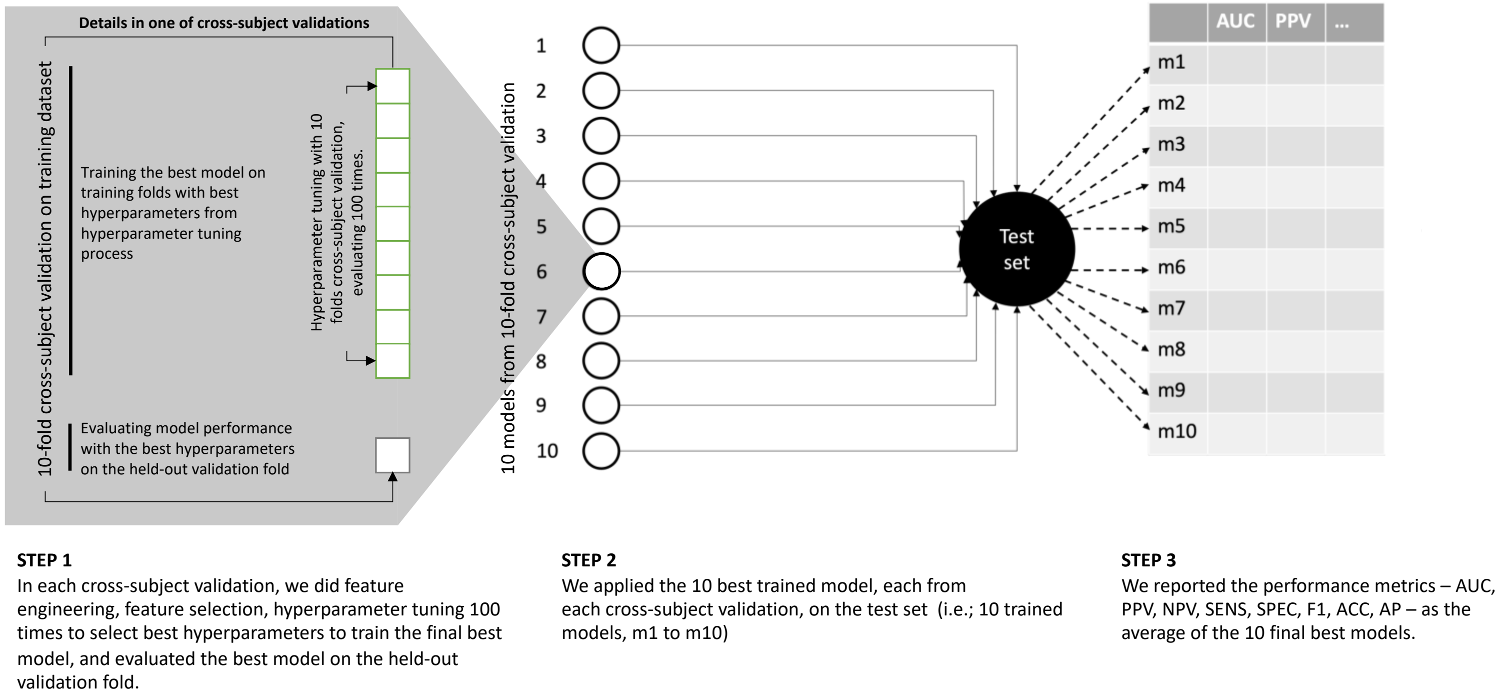

Supplementary Figure 6: Steps in model building and evaluation

Supplementary Table 1: Selected unique features for model week 4

Source feature	Feature importance	Estimate	Adjusted odds ratio	p-value	type	Known associations
**age_preg**	3.89	0.017295784	1.017446223	0.550880887	demo	Y
**SYSTOLIC BLOOD PRESSURE**	2.43	0.33475149	1.397593027	4.03249E-74	vital	Y
**DIASTOLIC BLOOD PRESSURE**	1.85	0.31477006	1.369944269	1.29053E-56	vital	Y
**African_American**	1.28	0.955756514	2.600637259	1.1267E-52	demo	N
**pe_hist**	1.17	2.45635387	11.66221198	3.7361E-154	demo	Y
**Caucasian**	1.15	-0.686828704	0.50316924	2.59001E-31	demo	N
**medicaid**	1.03	0.640257955	1.896970148	3.24087E-28	demo	N
**Other female genital disorders**	1.02	0.021272725	1.021500603	6.40031E-06	dx	N
**WEIGHT**	0.94	0.185615074	1.203958737	8.10297E-23	vital	Y
**Hispanic**	0.82	0.412908536	1.511206799	1.08281E-09	demo	N
**Asian**	0.68	-0.507282478	0.602129658	0.000111467	demo	N
**MEAN CORP. VOLUME**	0.61	-0.084442121	0.919024846	1.41535E-05	lab	N
**Contraceptive and procreative management**	0.6	0.00690972	1.006933647	0.229379453	dx	N
**TEMPERATURE**	0.52	-0.092326185	0.911807683	8.15368E-05	vital	N
**Propofol**	0.5	-0.011373243	0.988691188	0.35733058	rx	N
**PULSE**	0.49	0.197880047	1.218816184	1.99776E-16	vital	N
**Headache; including migraine**	0.42	0.047285439	1.048421227	1.61654E-14	dx	Y
**Menstrual disorders**	0.38	0.004744737	1.004756011	0.436202365	dx	N
**RBC BLOOD CELL**	0.37	0.081440293	1.084848443	0.002882815	lab	N

Supplementary Table 2: Selected unique features for model week 8

Source feature	Feature importance	Estimate	Adjusted odds ratio	p-value	type	Known associations
**SYSTOLIC BLOOD PRESSURE**	2.56	0.279953	1.323067	7.93E-27	vital	Y
**age_preg**	2.45	0.017296	1.017446	0.550881	demo	Y
**DIASTOLIC BLOOD PRESSURE**	1.98	0.367213	1.443705	1.46E-76	vital	Y
**African_American**	1.25	0.955757	2.600637	1.13E-52	demo	N
**pe_hist**	1.21	2.456354	11.66221	3.7E-154	demo	Y
**intrauterine fetal demise**	0.89	0.02407	1.024362	1.29E-21	dx	N
**Hispanic**	0.85	0.412909	1.511207	1.08E-09	demo	N
**WEIGHT**	0.81	0.247785	1.281185	2.04E-23	vital	Y
**medicaid**	0.77	0.640258	1.89697	3.24E-28	demo	N
**Caucasian**	0.75	-0.68683	0.503169	2.59E-31	demo	N
**VARICELLA-ZOSTER IGG**	0.59	-0.00482	0.995191	0.876679	lab	N
**RED DISTRIB. WIDTH**	0.51	0.04901	1.050231	0.037474	lab	N
**PLATELET**	0.49	-0.03393	0.966636	0.2227	lab	N
**MEAN CORP. VOLUME**	0.48	-0.09731	0.907273	2.92E-05	lab	N
**Asian**	0.47	-0.50728	0.60213	0.000111	demo	N
**MEAN PLT VOLUME**	0.44	0.088326	1.092344	0.001523	lab	N
**WHITE BLOOD CELL**	0.43	0.03073	1.031207	0.280015	lab	Y
**HEMOGLOBIN**	0.39	0.005777	1.005793	0.84231	lab	Y
**PULSE**	0.32	0.257451	1.293629	3.02E-26	vital	N
**MEAN CORP. HGB CONC.**	0.32	0.105064	1.110782	9.13E-05	lab	N
**HEMATOCRIT**	0.32	0.05731	1.058984	0.028526	lab	N
**RBC BLOOD CELL**	0.32	-0.03734	0.963344	0.188939	lab	N
**Other screening for suspected conditions (not mental disorders or infectious disease)**	0.32	0.012794	1.012876	5.4E-08	dx	N

Supplementary Table 3: Selected unique features for model week 12

Source feature	Feature importance	Estimate	Adjusted odds ratio	p-value	type	Known associations
**age_preg**	2.84	0.017303983	1.017455	0.550692	demo	Y
**pe_hist**	2.19	2.456335571	11.662	3.8E-154	demo	Y
**SYSTOLIC BLOOD PRESSURE**	2.09	0.322498107	1.380572	6.56E-32	vital	Y
**DIASTOLIC BLOOD PRESSURE**	1.82	0.418160206	1.519164	1.21E-93	vital	Y
**African_American**	1.58	0.955735195	2.600582	1.13E-52	demo	N
**Caucasian**	1.07	-0.686797208	0.503185	2.61E-31	demo	N
**WEIGHT**	0.95	0.335163704	1.398169	2.91E-39	vital	Y
**Other screening for suspected conditions (not mental disorders or infectious disease)**	0.68	0.005166863	1.00518	0.00067	dx	N
**MEAN CORP. VOLUME**	0.66	0.051727137	1.053088	0.032007	lab	N
**higher order multiplee**	0.66	0.03192773	1.032443	9.01E-07	dx	N
**Hispanic**	0.65	0.412886639	1.511174	1.09E-09	demo	N
**RED DISTRIB. WIDTH**	0.64	0.101008717	1.106286	5.17E-09	lab	N
**MEAN CORP. HGB CONC.**	0.63	-0.138477477	0.870683	1.06E-07	lab	N
**medicaid**	0.62	0.640315037	1.897078	3.21E-28	demo	N
**PH - DIPSTICK**	0.62	0.044755097	1.045772	0.115067	lab	N
**RBC BLOOD CELL**	0.6	0.067813999	1.070166	0.015482	lab	N
**MEAN CORP. HGB**	0.57	-0.043569692	0.957366	0.121727	lab	N
**PLATELET**	0.53	-0.07384261	0.928818	0.001703	lab	N
**Menstrual disorders**	0.53	-0.002196103	0.997806	0.32397	dx	N
**LYMPHOCYTE %**	0.53	0.205215813	1.22779	2.76E-15	lab	N
**MEAN PLT VOLUME**	0.51	0.042397733	1.043309	0.129841	lab	N
**PULSE**	0.5	0.115064183	1.121945	8.21E-06	vital	N
**Asian**	0.49	-0.50730215	0.602118	0.000111	demo	N
**Oxytocin**	0.47	-0.009023532	0.991017	0.638098	rx	N
**WHITE BLOOD CELL**	0.47	-0.065756363	0.936359	0.016343	lab	Y
**HEMOGLOBIN**	0.45	0.024006407	1.024297	0.405191	lab	Y
**Other pregnancy and delivery including normal**	0.42	0.010441012	1.010496	2.82E-13	dx	N
**VARICELLA-ZOSTER IGG**	0.42	-0.022978555	0.977283	0.537668	lab	N
**HEMATOCRIT**	0.4	0.292375893	1.339606	7.48E-27	lab	N
**QTC**	0.35	0.211634956	1.235697	1.2E-41	lab	N

Supplementary Table 4: Selected unique features for model week 16

Source feature	Feature importance	Estimate	Adjusted odds ratio	p-value	type	Known associations
**SYSTOLIC BLOOD PRESSURE**	2.96	0.37141	1.449777	1.07E-39	vital	Y
**age_preg**	2.8	0.017258	1.017408	0.551748	demo	Y
**pe_hist**	2.79	2.456262	11.66114	3.8E-154	demo	Y
**DIASTOLIC BLOOD PRESSURE**	2.22	0.446819	1.563331	4.6E-106	vital	Y
**African_American**	1.75	0.956069	2.60145	1.04E-52	demo	N
**twin pregnancy**	1.19	0.022797	1.023059	3.39E-09	dx	N
**WEIGHT**	1.11	0.221214	1.247591	8.21E-30	vital	Y
**Caucasian**	1	-0.68697	0.5031	2.52E-31	demo	N
**Hispanic**	0.83	0.413049	1.511419	1.07E-09	demo	N
**Other screening for suspected conditions (not mental disorders or infectious disease)**	0.8	0.004822	1.004834	0.000388	dx	N
**PULSE**	0.77	-0.03132	0.969161	0.275455	vital	N
**RED DISTRIB. WIDTH**	0.75	-0.07223	0.930316	0.004888	lab	N
**Headache; including migraine**	0.7	0.031587	1.032091	4.35E-26	dx	Y
**U-PROTEIN**	0.68	0.303927	1.355171	3.71E-72	lab	Y
**MEAN CORP. VOLUME**	0.67	-0.14404	0.86585	1.18E-12	lab	N
**PLATELET**	0.66	0.034389	1.034987	0.222239	lab	N
**MEAN CORP. HGB**	0.66	-0.12481	0.882668	2.9E-07	lab	N
**gestational hypertension**	0.63	0.064516	1.066643	9.82E-87	dx	Y
**LYMPHOCYTE %**	0.61	0.209727	1.233342	1.59E-15	lab	N
**WHITE BLOOD CELL**	0.59	0.0764	1.079394	0.000328	lab	Y
**medicaid**	0.54	0.640293	1.897036	3.22E-28	demo	N
**PH - DIPSTICK**	0.53	0.080683	1.084027	0.003703	lab	N
**HEMOGLOBIN**	0.53	0.043851	1.044827	0.121799	lab	Y
**HEMATOCRIT**	0.52	-0.0674	0.934819	0.008571	lab	N
**Other female genital disorders**	0.52	0.012553	1.012632	4.02E-10	dx	N
**MEAN CORP. HGB CONC.**	0.51	-0.08618	0.917432	0.001618	lab	N
**Oxytocin**	0.48	-0.00914	0.990898	0.632377	rx	N
**MEAN PLT VOLUME**	0.47	0.103712	1.109281	4.5E-06	lab	N
**Residual codes; unclassified**	0.45	0.010302	1.010355	2.24E-09	dx	N
**RESPIRATIONS**	0.41	-0.04347	0.957462	0.076876	vital	N
**RBC BLOOD CELL**	0.4	-0.09872	0.905994	0.000207	lab	N
**MONOCYTE %**	0.39	0.239263	1.270313	3.25E-21	lab	N
**URIC ACID-BLD**	0.39	0.246033	1.278941	5.12E-96	lab	N
**VARICELLA-ZOSTER IGG**	0.38	-0.02732	0.973052	0.480327	lab	N
**CREATININE-SERUM**	0.38	0.019701	1.019896	0.032606	lab	N
**LDH-BLD**	0.36	0.248244	1.281773	2.01E-76	lab	N

Supplementary Table 5: Selected unique features for model week 20

Source feature	Feature importance	Estimate	Adjusted odds ratio	p-value	type	Known associations
**SYSTOLIC BLOOD PRESSURE**	2.57	0.398581	1.489709	1.34E-43	vital	Y
**age_preg**	1.98	0.016979	1.017124	0.558193	demo	Y
**pe_hist**	1.46	2.455658	11.6541	4.6E-154	demo	Y
**African_American**	1.32	0.957462	2.605076	7.47E-53	demo	N
**DIASTOLIC BLOOD PRESSURE**	1.31	0.462023	1.587282	1.9E-114	vital	Y
**twin pregnancy**	1.06	0.02177	1.022009	2.38E-11	dx	N
**U-PROTEIN**	0.92	0.360663	1.43428	2.2E-106	lab	Y
**Caucasian**	0.82	-0.68807	0.502545	2.03E-31	demo	N
**MEAN CORP. VOLUME**	0.79	-0.10892	0.896802	7.08E-06	lab	N
**Other screening for suspected conditions (not mental disorders or infectious disease)**	0.77	0.006107	1.006126	1.5E-06	dx	N
**HEMOGLOBIN**	0.69	-0.04862	0.952542	0.077445	lab	Y
**WEIGHT**	0.63	0.244241	1.276652	9.38E-33	vital	Y
**MEAN CORP. HGB CONC.**	0.55	-0.00084	0.999162	0.976913	lab	N
**PULSE**	0.5	0.08081	1.084165	0.003572	vital	N
**MEAN CORP. HGB**	0.47	0.028392	1.028799	0.326841	lab	N
**Headache; including migraine**	0.46	0.029865	1.030315	2.31E-30	dx	Y
**unspecified GDM**	0.46	0.028787	1.029205	5.63E-19	dx	N
**RBC BLOOD CELL**	0.45	0.072182	1.074851	0.008708	lab	N
**WHITE BLOOD CELL**	0.44	0.027093	1.027464	0.344867	lab	Y
**MEAN PLT VOLUME**	0.43	0.076747	1.079768	0.003209	lab	N
**HEMATOCRIT**	0.43	0.014924	1.015036	0.605565	lab	N
**Residual codes; unclassified**	0.41	0.009419	1.009463	3.86E-10	dx	N
**URIC ACID-BLD**	0.4	0.286711	1.332039	4.5E-131	lab	N
**RED DISTRIB. WIDTH**	0.39	0.037371	1.038078	0.043907	lab	N
**VARICELLA-ZOSTER IGG**	0.38	-0.0293	0.971121	0.456334	lab	N
**ALT(SGPT)**	0.37	0.032791	1.033335	0.110267	lab	N
**PLATELET**	0.37	0.008211	1.008245	0.63313	lab	N
**LYMPHOCYTE %**	0.37	0.280372	1.323622	2.13E-27	lab	N
**Hispanic**	0.36	0.41458	1.513735	9.29E-10	demo	N
**medicaid**	0.35	0.641008	1.898393	2.81E-28	demo	N
**U-CREATININE (TIMED)**	0.35	0.196095	1.216642	7.36E-59	lab	N
**Oxytocin**	0.34	-0.00934	0.990704	0.62346	rx	N
**NEUTROPHIL %**	0.34	0.292534	1.339818	3.26E-26	lab	N
**PH - DIPSTICK**	0.33	-0.04516	0.955841	0.117388	lab	N
**LDH-BLD**	0.3	0.277445	1.319753	3.01E-92	lab	N

Supplementary Table 6: Selected unique features for model week 22

Source feature	Feature importance	Estimate	Adjusted odds ratio	p-value	type	Known associations
**age_preg**	4.39	0.016656	1.016795	0.565702	demo	Y
**SYSTOLIC BLOOD PRESSURE**	3.51	0.416524	1.516681	2.48E-46	vital	Y
**pe_hist**	3.5	2.454486	11.64045	6.4E-154	demo	Y
**DIASTOLIC BLOOD PRESSURE**	2.78	0.486965	1.627369	2.4E-127	vital	Y
**African_American**	2.59	0.960299	2.612478	3.77E-53	demo	N
**twin pregnancy**	2.1	0.021054	1.021277	4.23E-12	dx	N
**RBC BLOOD CELL**	1.69	0.039529	1.040321	0.170245	lab	N
**Other screening for suspected conditions (not mental disorders or infectious disease)**	1.63	0.00736	1.007388	2.96E-09	dx	N
**U-PROTEIN**	1.59	0.382911	1.466547	1.2E-120	lab	Y
**WEIGHT**	1.47	0.234509	1.264288	1.14E-29	vital	Y
**RED DISTRIB. WIDTH**	1.18	0.107171	1.113124	3.27E-08	lab	N
**MEAN CORP. VOLUME**	1.16	-0.06277	0.939157	0.01369	lab	N
**Caucasian**	1.16	-0.68975	0.5017	1.45E-31	demo	N
**gestational hypertension**	1.1	0.059175	1.060961	1.4E-126	dx	Y
**MEAN PLT VOLUME**	1.09	-0.00975	0.990299	0.736049	lab	N
**MEAN CORP. HGB CONC.**	1.08	-0.05207	0.949263	0.065408	lab	N
**LYMPHOCYTE %**	0.98	0.28627	1.331452	2.2E-28	lab	N
**Oxytocin**	0.98	-0.00988	0.990171	0.600301	rx	N
**unspecified GDM**	0.96	0.026182	1.026528	6.24E-18	dx	N
**intrauterine fetal demise**	0.91	0.018371	1.018541	4.52E-45	dx	N
**WHITE BLOOD CELL**	0.9	-0.01607	0.984055	0.585434	lab	Y
**O2 SATURATION**	0.89	-0.02102	0.9792	0.60592	vital	N
**PH - DIPSTICK**	0.87	-0.11351	0.892693	5.21E-05	lab	N
**advanced maternal age**	0.82	0.003775	1.003782	0.025884	dx	Y
**NEUTROPHIL #**	0.82	0.273371	1.314388	3.94E-27	lab	N
**PULSE**	0.79	0.190048	1.209308	1.63E-14	vital	N
**Headache; including migraine**	0.78	0.029206	1.029637	1.34E-31	dx	Y
**VARICELLA-ZOSTER IGG**	0.77	-0.02881	0.9716	0.461866	lab	N
**ALT(SGPT)**	0.77	0.062956	1.06498	0.039941	lab	N
**MEAN CORP. HGB**	0.75	-0.11052	0.895364	2.8E-06	lab	N
**LDH-BLD**	0.74	0.291921	1.338997	4.54E-97	lab	N
**MONOCYTE %**	0.74	0.275806	1.317592	9.58E-28	lab	N
**HEMATOCRIT**	0.74	0.045964	1.047037	0.099382	lab	N
**SPEC GRAVITY-DIPSTICK**	0.73	0.009235	1.009278	0.909824	lab	N
**Other pregnancy and delivery including normal**	0.69	0.010137	1.010189	5.23E-16	dx	N
**HEMOGLOBIN**	0.68	0.007741	1.007771	0.788199	lab	Y
**medicaid**	0.67	0.641573	1.899467	2.53E-28	demo	N
**Residual codes; unclassified**	0.64	0.009026	1.009067	7.82E-11	dx	N
**POTASSIUMBLD**	0.64	0.272633	1.313418	1.37E-44	lab	N
**HEIGHT**	0.63	0.294155	1.341992	1.52E-26	vital	N

Supplementary Table 7: Selected unique features for model week 24

Source feature	Feature importance	Estimate	Adjusted odds ratio	p-value	type	Known associations
**age_preg**	4.38	0.017884	1.018045	0.537622	demo	Y
**pe_hist**	3.77	2.453614	11.6303	8.5E-154	demo	Y
**SYSTOLIC BLOOD PRESSURE**	3.72	0.583687	1.792637	3.3E-215	vital	Y
**DIASTOLIC BLOOD PRESSURE**	3.34	0.514945	1.673547	3.2E-141	vital	Y
**African_American**	2.61	0.960672	2.613452	4.27E-53	demo	N
**twin pregnancy**	2.37	0.021319	1.021548	8.74E-14	dx	N
**Other screening for suspected conditions (not mental disorders or infectious disease)**	2.26	0.008617	1.008654	2.73E-12	dx	N
**U-PROTEIN**	1.95	0.405699	1.500351	1.5E-135	lab	Y
**WEIGHT**	1.8	0.281771	1.325476	2.1E-56	vital	Y
**MEAN CORP. HGB**	1.76	0.068021	1.070388	0.014552	lab	N
**unspecified GDM**	1.72	0.025057	1.025374	7.48E-20	dx	N
**WHITE BLOOD CELL**	1.67	0.170879	1.186347	1.72E-13	lab	Y
**MEAN CORP. VOLUME**	1.4	-0.09803	0.906626	2.23E-05	lab	N
**FETAL FIBRONECTIN**	1.38	0.083655	1.087254	0.0001	lab	N
**gestational hypertension**	1.35	0.058596	1.060346	4E-142	dx	Y
**intrauterine fetal demise**	1.22	0.017982	1.018145	7.08E-46	dx	N
**RBC BLOOD CELL**	1.17	0.031786	1.032296	0.266075	lab	N
**Caucasian**	1.15	-0.69007	0.501541	1.42E-31	demo	N
**Headache; including migraine**	1.11	0.029542	1.029983	3.23E-36	dx	Y
**MONOCYTE %**	1.06	0.259413	1.296169	1.45E-22	lab	N
**RED DISTRIB. WIDTH**	1	0.04111	1.041967	0.1122	lab	N
**HEMATOCRIT**	0.97	0.017045	1.017191	0.556662	lab	N
**HEMOGLOBIN**	0.97	0.047942	1.04911	0.100017	lab	Y
**LDH-BLD**	0.97	0.304929	1.356528	1.9E-104	lab	N
**Oxytocin**	0.97	0.017167	1.017315	0.191914	rx	N
**MEAN PLT VOLUME**	0.96	0.054641	1.056162	0.054351	lab	N
**ALT(SGPT)**	0.94	0.085953	1.089755	0.000419	lab	N
**MEAN CORP. HGB CONC.**	0.9	-0.08213	0.921149	0.003598	lab	N
**LYMPHOCYTE %**	0.88	0.2976	1.346623	1.8E-30	lab	N
**RESPIRATIONS**	0.87	0.041982	1.042876	0.028083	vital	N
**PH - DIPSTICK**	0.84	-0.01745	0.9827	0.545635	lab	N
**PULSE**	0.77	0.095485	1.100193	0.000594	vital	N
**VARICELLA-ZOSTER IGG**	0.73	-0.02805	0.972343	0.470532	lab	N
**nonreassuring fetal status**	0.71	0.00258	1.002583	0.038058	dx	N
**POTASSIUMBLD**	0.7	0.285293	1.330151	1.58E-48	lab	N
**O2 SATURATION**	0.7	-0.02916	0.971262	0.496507	vital	N
**Contraceptive and procreative management**	0.7	0.005034	1.005046	0.020523	dx	N
**NEUTROPHIL #**	0.65	0.2875	1.333091	8.19E-30	lab	N

Supplementary Table 8: Selected unique features for model week 26

Source feature	Feature importance	Estimate	Adjusted odds ratio	p-value	type	Known associations
**SYSTOLIC BLOOD PRESSURE**	3.6	0.456552	1.578622	5.44E-52	vital	Y
**age_preg**	2.76	0.015092	1.015207	0.604166	demo	Y
**DIASTOLIC BLOOD PRESSURE**	2.32	0.533845	1.705478	1.8E-151	vital	Y
**pe_hist**	2.23	2.449529	11.58289	1E-151	demo	Y
**African_American**	1.61	0.964382	2.623166	4.17E-53	demo	N
**twin pregnancy**	1.38	0.020733	1.020949	6.93E-14	dx	N
**U-PROTEIN**	1.32	0.42952	1.53652	1.9E-150	lab	Y
**WEIGHT**	1.15	-0.12492	0.88257	5.25E-09	vital	Y
**gestational hypertension**	1.08	0.057529	1.059216	9.2E-170	dx	Y
**Other screening for suspected conditions (not mental disorders or infectious disease)**	1.02	0.008511	1.008547	4.58E-12	dx	N
**FETAL FIBRONECTIN**	0.94	0.116482	1.123538	3.56E-08	lab	N
**MEAN CORP. VOLUME**	0.91	-0.05542	0.946092	0.030968	lab	N
**Oxytocin**	0.85	-0.01024	0.989808	0.577733	rx	N
**Caucasian**	0.85	-0.69023	0.501463	2.21E-31	demo	N
**URIC ACID-BLD**	0.81	0.349676	1.418607	2.1E-189	lab	N
**Headache; including migraine**	0.81	0.029708	1.030154	2.37E-40	dx	Y
**MEAN CORP. HGB**	0.78	-0.10406	0.901175	3.12E-05	lab	N
**MEAN PLT VOLUME**	0.76	0.065406	1.067592	0.016785	lab	N
**HEMATOCRIT**	0.74	0.004679	1.00469	0.871946	lab	N
**unspecified GDM**	0.68	0.019413	1.019602	1.59E-16	dx	N
**RBC BLOOD CELL**	0.65	0.004744	1.004755	0.870551	lab	N
**LDH-BLD**	0.62	0.328358	1.388686	1.6E-120	lab	N
**MEAN CORP. HGB CONC.**	0.61	-0.08447	0.919002	0.002656	lab	N
**MONOCYTE %**	0.6	0.254296	1.289554	3.9E-21	lab	N
**PLATELET**	0.59	-0.01539	0.984729	0.850649	lab	N
**HEMOGLOBIN**	0.58	0.036373	1.037043	0.214696	lab	Y
**WHITE BLOOD CELL**	0.54	0.000882	1.000882	0.975803	lab	Y
**PULSE**	0.53	-0.1237	0.883647	8.1E-07	vital	N
**ALT(SGPT)**	0.52	0.178881	1.195878	4.15E-24	lab	N
**RED DISTRIB. WIDTH**	0.5	0.044961	1.045987	0.080831	lab	N
**NEUTROPHIL %**	0.47	0.329653	1.390485	1.12E-30	lab	N
**medicaid**	0.46	0.646144	1.908169	1.73E-28	demo	N
**Abdominal pain**	0.44	0.014128	1.014228	2.19E-16	dx	N
**O2 SATURATION**	0.44	-0.00044	0.999565	0.987891	vital	N
**CREATININE-SERUM**	0.42	0.016216	1.016348	0.056421	lab	N
**intrauterine fetal demise**	0.42	0.017049	1.017195	1.88E-43	dx	N

Supplementary Table 9: Selected unique features for model week 28

Source feature	Feature importance	Estimate	Adjusted odds ratio	p-value	type	Known associations
**SYSTOLIC BLOOD PRESSURE**	4.41	0.512214682	1.668983372	4.02269E-61	vital	Y
**pe_hist**	2.71	2.443039566	11.50796686	2.3127E-149	demo	Y
**age_preg**	2.52	0.013380352	1.01347027	0.646690228	demo	Y
**DIASTOLIC BLOOD PRESSURE**	2.22	0.544736696	1.724154345	1.3919E-154	vital	Y
**gestational hypertension**	2.08	0.058763921	1.060524843	1.5225E-217	dx	Y
**FETAL FIBRONECTIN**	1.86	0.132172366	1.141305024	7.78058E-10	lab	N
**African_American**	1.78	0.958251414	2.607133688	5.66104E-52	demo	N
**twin pregnancy**	1.74	0.021159419	1.021384867	5.39075E-16	dx	N
**MEAN CORP. HGB CONC.**	1.37	-0.025549147	0.974774471	0.379547025	lab	N
**URIC ACID-BLD**	1.23	0.393031132	1.48146451	1.5065E-239	lab	N
**WEIGHT**	1.22	0.530978049	1.700594761	1.95923E-81	vital	Y
**Other screening for suspected conditions (not mental disorders or infectious disease)**	1.18	0.00898057	1.009021016	2.55257E-13	dx	N
**U-PROTEIN**	1.1	0.459908054	1.583928342	1.7393E-170	lab	Y
**unspecified GDM**	1.05	0.01940885	1.019598426	2.7706E-25	dx	N
**MEAN CORP. HGB**	0.99	-0.205194778	0.814488656	3.91702E-22	lab	N
**Oxytocin**	0.84	0.006141983	1.006160884	0.675306733	rx	N
**Headache; including migraine**	0.83	0.030175582	1.030635479	3.88742E-46	dx	Y
**ALT(SGPT)**	0.82	0.104839416	1.110532262	2.83936E-05	lab	N
**PULSE**	0.8	-0.041640114	0.959214926	0.147121222	vital	N
**LDH-BLD**	0.79	0.363703746	1.438647946	1.0446E-146	lab	N
**RBC BLOOD CELL**	0.78	-0.083516953	0.919875492	0.003256881	lab	N
**GLUCOSE 1 HR**	0.74	0.291362656	1.338249821	4.9838E-30	lab	N
**MEAN CORP. VOLUME**	0.74	-0.224295204	0.799079208	4.78285E-26	lab	N
**Caucasian**	0.67	-0.689189801	0.50198261	3.86791E-31	demo	N
**RED DISTRIB. WIDTH**	0.64	0.033679462	1.034253036	0.121623296	lab	N
**HEMOGLOBIN**	0.63	0.027761469	1.028150409	0.345772849	lab	Y
**RESPIRATIONS**	0.63	-0.019629274	0.980562125	0.50688978	vital	N
**PretermL**	0.62	0.013270847	1.013359296	1.05858E-18	dx	N
**MEAN PLT VOLUME**	0.62	0.049258796	1.050492179	0.090528959	lab	N
**MONOCYTE %**	0.61	0.318910277	1.375627894	3.24204E-34	lab	N
**Allergic reactions**	0.58	0.014309355	1.014412224	1.38983E-16	dx	N
**WHITE BLOOD CELL**	0.58	0.106001884	1.111823971	3.84622E-05	lab	Y
**HEMATOCRIT**	0.58	-0.006437887	0.993582792	0.825233055	lab	N
**PLATELET**	0.57	0.010838536	1.010897486	0.290237264	lab	N

Supplementary Table 10: Selected unique features for model week 30

Source feature	Feature importance	Estimate	Adjusted odds ratio	p-value	type	Known associations
**SYSTOLIC BLOOD PRESSURE**	3.58	0.538657	1.713704	1.1E-63	vital	Y
**pe_hist**	2.8	2.437368	11.44288	2.5E-146	demo	Y
**age_preg**	2.25	0.016709	1.016849	0.570043	demo	Y
**twin pregnancy**	1.8	0.020292	1.020499	6.86E-16	dx	N
**gestational hypertension**	1.8	0.059017	1.060793	3.3E-273	dx	Y
**FETAL FIBRONECTIN**	1.67	0.155409	1.168135	6.81E-13	lab	N
**DIASTOLIC BLOOD PRESSURE**	1.59	0.545168	1.724898	5.5E-152	vital	Y
**African_American**	1.54	0.95883	2.608642	3.32E-51	demo	N
**URIC ACID-BLD**	1.39	0.428149	1.534415	3.1E-274	lab	N
**Other screening for suspected conditions (not mental disorders or infectious disease)**	1.18	0.009458	1.009503	1.53E-14	dx	N
**MEAN CORP. VOLUME**	0.99	-0.22836	0.795839	2.53E-26	lab	N
**Headache; including migraine**	0.98	0.030143	1.030602	1.58E-50	dx	Y
**WEIGHT**	0.96	0.239602	1.270744	1.83E-27	vital	Y
**U-PROTEIN**	0.89	0.495144	1.640735	3.5E-194	lab	Y
**RED DISTRIB. WIDTH**	0.79	0.059003	1.060778	0.004377	lab	N
**MEAN CORP. HGB CONC.**	0.74	-0.08015	0.922978	0.004978	lab	N
**Oxytocin**	0.73	0.005843	1.00586	0.680944	rx	N
**PLATELET**	0.73	0.01577	1.015895	0.177961	lab	N
**Caucasian**	0.7	-0.68421	0.50449	2.5E-30	demo	N
**intrauterine fetal demise**	0.69	0.018431	1.018602	1.97E-57	dx	N
**MEAN CORP. HGB**	0.64	-0.23514	0.790458	1.12E-24	lab	N
**Other pregnancy and delivery including normal**	0.62	0.004259	1.004268	0.000314	dx	N
**PULSE**	0.6	-0.07689	0.925994	0.005543	vital	N
**MEAN PLT VOLUME**	0.6	0.036115	1.036775	0.216423	lab	N
**HEMATOCRIT**	0.59	-0.01725	0.982903	0.557052	lab	N
**RBC BLOOD CELL**	0.58	0.175926	1.19235	1.18E-10	lab	N
**WHITE BLOOD CELL**	0.57	0.10515	1.110877	7.63E-05	lab	Y
**O2 SATURATION**	0.53	-0.03036	0.970099	0.668428	vital	N
**LYMPHOCYTE %**	0.52	0.342798	1.408884	3.11E-37	lab	N
**LDH-BLD**	0.5	0.399441	1.490992	1.4E-173	lab	N
**FIBRINOGEN**	0.5	0.45427	1.575024	4.6E-294	lab	N
**ALT(SGPT)**	0.5	0.106334	1.112193	1.84E-05	lab	N
**HEMOGLOBIN**	0.49	0.035909	1.036562	0.219787	lab	Y
**ALK PHOSPHATASE, BLD**	0.48	0.292409	1.339651	2.99E-62	lab	N

Supplementary Table 11: Selected unique features for model week 32

Source feature	Feature importance	Estimate	Adjusted odds ratio	p-value	type	Known associations
**SYSTOLIC BLOOD PRESSURE**	4.65	0.60619	1.833432	4.83E-72	vital	Y
**pe_hist**	2.62	2.427387	11.32924	3.2E-141	demo	Y
**gestational hypertension**	2.46	0.058112	1.059834	0	dx	Y
**DIASTOLIC BLOOD PRESSURE**	1.89	0.642766	1.901734	2.36E-81	vital	Y
**age_preg**	1.89	0.007904	1.007936	0.790512	demo	Y
**twin pregnancy**	1.64	0.018471	1.018643	3.95E-13	dx	N
**FETAL FIBRONECTIN**	1.49	0.181901	1.199496	5.52E-17	lab	N
**African_American**	1.44	0.936165	2.550182	2.63E-47	demo	N
**MEAN CORP. HGB**	1.38	-0.16166	0.850733	1.11E-12	lab	N
**Headache; including migraine**	1.18	0.031175	1.031666	6.64E-62	dx	Y
**Other screening for suspected conditions (not mental disorders or infectious disease)**	1.14	0.010179	1.010231	2.06E-16	dx	N
**WEIGHT**	1.11	0.33154	1.393112	4.11E-68	vital	Y
**U-PROTEIN**	0.88	0.561581	1.753443	1.2E-247	lab	Y
**unspecified GDM**	0.83	0.016284	1.016417	1.54E-25	dx	N
**MEAN PLT VOLUME**	0.82	-0.07591	0.926902	0.010609	lab	N
**FIBRINOGEN**	0.81	0.527481	1.694658	0	lab	N
**PULSE**	0.81	-0.05776	0.943876	0.042852	vital	N
**URIC ACID-BLD**	0.8	0.495093	1.640651	0	lab	N
**MEAN CORP. HGB CONC.**	0.79	-0.14499	0.865031	2.3E-07	lab	N
**O2 SATURATION**	0.77	-0.00084	0.999157	0.977392	vital	N
**PLATELET**	0.77	0.87738	2.404591	0.004874	lab	N
**RED DISTRIB. WIDTH**	0.76	0.056718	1.058357	0.003923	lab	N
**Essential hypertension**	0.71	0.05644	1.058063	8.3E-147	dx	Y
**MEAN CORP. VOLUME**	0.67	-0.13314	0.875345	9.54E-06	lab	N
**PretermL**	0.67	0.013712	1.013807	5.51E-29	dx	N
**WHITE BLOOD CELL**	0.66	-0.01806	0.982104	0.534089	lab	Y
**MONOCYTE %**	0.56	0.34719	1.415086	3.25E-34	lab	N
**HEMOGLOBIN**	0.56	-0.04404	0.956912	0.072439	lab	Y
**RESPIRATIONS**	0.55	-0.01313	0.98696	0.67305	vital	N
**LYMPHOCYTE %**	0.55	0.347576	1.415631	9.78E-36	lab	N
**HEMATOCRIT**	0.55	-0.04825	0.952894	0.092603	lab	N
**PH - DIPSTICK**	0.54	-0.12553	0.882028	1.12E-05	lab	N
**EOSINOPHIL %**	0.54	0.154687	1.167293	2.86E-14	lab	N
**ALK PHOSPHATASE, BLD**	0.5	0.289199	1.335357	7.44E-62	lab	N

Supplementary Table 12: Selected unique features for model week 34

Source feature	Feature importanc e	Estimate	Adjusted odds ratio	p-value	type	Known associations
**SYSTOLIC BLOOD PRESSURE**	5.3	0.653821	1.922875	1.43E-72	vital	Y
**gestational hypertension**	3.23	0.056492	1.058118	0	dx	Y
**pe_hist**	2.51	2.420706	11.2538	1.7E-131	demo	Y
**age_preg**	2.34	0.008315	1.008349	0.78699	demo	Y
**FIBRINOGEN**	1.92	0.588711	1.801665	0	lab	N
**DIASTOLIC BLOOD PRESSURE**	1.71	0.555188	1.742268	4.5E-141	vital	Y
**WHITE BLOOD CELL**	1.65	0.111964	1.118473	4.54E-05	lab	Y
**Other pregnancy and delivery including normal**	1.49	-0.00156	0.99844	0.221939	dx	N
**URIC ACID-BLD**	1.44	0.548969	1.731467	0	lab	N
**U-PROTEIN**	1.34	0.609215	1.838987	2.1E-270	lab	Y
**large for GA**	1.33	0.010029	1.01008	4.09E-05	dx	N
**Headache; including migraine**	1.29	0.030646	1.03112	3.7E-61	dx	Y
**WEIGHT**	1.28	0.174777	1.190981	4.8E-15	vital	Y
**MEAN CORP. HGB**	1.24	-0.15373	0.857506	1.51E-10	lab	N
**HEMOGLOBIN**	1.2	-0.08934	0.914536	0.002092	lab	Y
**MEAN CORP. VOLUME**	1.05	-0.08684	0.916828	0.000778	lab	N
**twin pregnancy**	1.03	0.020257	1.020463	1.27E-15	dx	N
**HEMATOCRIT**	1	0.010178	1.01023	0.740753	lab	N
**chronic hypertension**	0.94	0.056093	1.057696	1.5E-197	dx	Y
**African_American**	0.9	0.934435	2.545776	4.75E-44	demo	N
**PLATELET**	0.86	4.172745	64.89333	3.45E-09	lab	Y
**fibroids in pregnancy**	0.86	0.016304	1.016437	3.42E-08	dx	N
**FETAL FIBRONECTIN**	0.85	0.215031	1.239901	1.05E-22	lab	N
**RED DISTRIB. WIDTH**	0.85	-0.00717	0.992859	0.812497	lab	N
**RESPIRATIONS**	0.79	0.025386	1.025711	0.307249	vital	N
**Other screening for suspected conditions (not mental disorders or infectious disease)**	0.77	0.00906	1.009101	8.12E-13	dx	N
**MONOCYTE %**	0.75	0.393142	1.481628	5.4E-45	lab	N
**PULSE**	0.73	0.1139	1.12064	4.98E-05	vital	N
**PretermL**	0.71	0.013535	1.013627	1.38E-30	dx	N
**MEAN PLT VOLUME**	0.67	0.142306	1.152929	3.63E-07	lab	N
**GLUCOSE 1 HR**	0.66	0.312527	1.366875	1.8E-28	lab	N
**ALK PHOSPHATASE, BLD**	0.61	0.283027	1.327141	4.29E-65	lab	N
**MEAN CORP. HGB CONC.**	0.58	0.023209	1.023481	0.447844	lab	N
**HEIGHT**	0.57	0.418862	1.52023	8.49E-41	vital	N
**EOSINOPHIL %**	0.56	0.15872	1.17201	2.09E-14	lab	N
**U-CREATININE (TIMED)**	0.55	0.332537	1.394501	2.3E-143	lab	N
**Essential hypertension**	0.53	0.054599	1.056117	9.5E-147	dx	Y
**RBC BLOOD CELL**	0.53	-0.16819	0.84519	4.11E-09	lab	N

Supplementary Table 13: Selected unique features for model week 35

Source feature	Feature importance	Estimate	Adjusted odds ratio	p-value	type	Known associations
**SYSTOLIC BLOOD PRESSURE**	3.69	0.684297	1.982377	2.19E-72	vital	Y
**gestational hypertension**	2.11	0.054435	1.055944	0	dx	Y
**pe_hist**	1.77	2.44342	11.51234	4.3E-130	demo	Y
**age_preg**	1.51	-0.00437	0.995637	0.889579	demo	Y
**FIBRINOGEN**	1.47	0.631588	1.880595	0	lab	N
**DIASTOLIC BLOOD PRESSURE**	1.38	0.577832	1.782171	2.3E-148	vital	Y
**MEAN CORP. VOLUME**	1.04	-0.24674	0.781341	1.13E-24	lab	N
**URIC ACID-BLD**	1.02	0.580663	1.787224	0	lab	N
**PLATELET**	1.01	-0.03824	0.962481	0.756975	lab	N
**MEAN PLT VOLUME**	0.99	0.014146	1.014246	0.65429	lab	N
**U-PROTEIN**	0.97	0.64151	1.899347	5.5E-284	lab	Y
**African_American**	0.91	0.93502	2.547264	4.96E-42	demo	N
**chronic hypertension**	0.85	0.054726	1.056251	4.5E-189	dx	Y
**WEIGHT**	0.8	0.125164	1.133334	1.93E-07	vital	Y
**Headache; including migraine**	0.78	0.03261	1.033147	8.31E-75	dx	Y
**WHITE BLOOD CELL**	0.73	0.026192	1.026538	0.394295	lab	Y
**MEAN CORP. HGB**	0.69	-0.09856	0.906139	0.000864	lab	N
**large for GA**	0.65	0.010398	1.010452	1.26E-05	dx	N
**fibroids in pregnancy**	0.64	0.018256	1.018423	2.05E-10	dx	N
**RED DISTRIB. WIDTH**	0.64	0.072813	1.07553	0.00257	lab	N
**Other ear and sense organ disorders**	0.62	0.024606	1.024912	5.14E-08	dx	N
**MEAN CORP. HGB CONC.**	0.58	-0.00154	0.998461	0.961018	lab	N
**HEMATOCRIT**	0.57	-0.06992	0.932471	0.020119	lab	N
**O2 SATURATION**	0.54	0.003124	1.003129	0.898801	vital	N
**PH - DIPSTICK**	0.51	-0.18519	0.830948	9.56E-10	lab	N
**RBC BLOOD CELL**	0.49	-0.05373	0.947691	0.065065	lab	N
**Other pregnancy and delivery including normal**	0.49	-0.00217	0.997834	0.110438	dx	N
**in vitro fertilization**	0.49	0.024409	1.024709	4.24E-13	dx	N
**Caucasian**	0.46	-0.66319	0.515206	3.35E-25	demo	N
**EOSINOPHIL %**	0.44	0.163849	1.178036	5.79E-15	lab	N
**HEMOGLOBIN**	0.41	0.018711	1.018887	0.52783	lab	Y
**obesity In pregnancy**	0.39	0.030777	1.031256	8.41E-52	dx	Y
**Residual codes; unclassified**	0.39	0.010444	1.010499	1.54E-19	dx	N
**LYMPHOCYTE %**	0.38	0.416495	1.516636	4.2E-47	lab	N
**RESPIRATIONS**	0.38	0.006189	1.006209	0.833065	vital	N
**PULSE**	0.38	0.026296	1.026645	0.400374	vital	N
**ALT(SGPT)**	0.37	0.15643	1.169329	8.29E-10	lab	N
**SPEC GRAVITY-DIPSTICK**	0.35	-0.02679	0.973568	0.892581	lab	N
MONOCYTE #	0.34	0.433823	1.543145	4.59E-53	lab	N

Supplementary Table 14: Selected unique features for model week 36

Source feature	Feature importance	Estimate	Adjusted odds ratio	p-value	type	Known associations
**SYSTOLIC BLOOD PRESSURE**	3.29	0.72831	2.071576	3.44E-71	vital	Y
**gestational hypertension**	2.34	0.053564	1.055024	0	dx	Y
**U-PROTEIN**	2.13	0.683811	1.981414	2.9E-298	lab	Y
**DIASTOLIC BLOOD PRESSURE**	2.11	0.544372	1.723526	1.2E-122	vital	Y
**pe_hist**	1.83	2.452551	11.61795	2.9E-121	demo	Y
**age_preg**	1.75	-0.02762	0.972756	0.400237	demo	Y
**FIBRINOGEN**	1.7	0.671959	1.95807	0	lab	N
**chronic hypertension**	1.57	0.054727	1.056253	9.6E-191	dx	Y
**Other pregnancy and delivery including normal**	1.51	-0.00483	0.995183	0.000855	dx	N
**PULSE**	1.42	0.239107	1.270114	5.27E-18	vital	N
**URIC ACID-BLD**	1.4	0.6104	1.841167	0	lab	N
**WEIGHT**	1.28	0.326035	1.385464	2.75E-54	vital	Y
**PLATELET**	0.91	7.674223	2152.151	2.12E-16	lab	N
**Other ear and sense organ disorders**	0.81	0.02456	1.024865	1.87E-07	dx	N
**RESPIRATIONS**	0.78	-0.03537	0.965248	0.17836	vital	N
**MEAN PLT VOLUME**	0.76	0.676362	1.96671	5.88E-67	lab	N
**EOSINOPHIL %**	0.74	0.165792	1.180327	1.75E-14	lab	N
**MEAN CORP. HGB**	0.74	-0.07098	0.931483	0.018216	lab	N
**Abdominal pain**	0.67	0.011784	1.011853	1.36E-12	dx	N
**Headache; including migraine**	0.67	0.031655	1.032161	4.8E-67	dx	Y
**Residual codes; unclassified**	0.65	0.010121	1.010172	2.22E-17	dx	N
**O2 SATURATION**	0.65	-0.00594	0.994077	0.878103	vital	N
**WHITE BLOOD CELL**	0.65	0.134475	1.143936	8.68E-06	lab	Y
**TEMPERATURE**	0.64	-0.03578	0.964851	0.249146	vital	N
**RED DISTRIB. WIDTH**	0.62	-0.03069	0.969772	0.33706	lab	N
**HEMATOCRIT**	0.62	0.038145	1.038882	0.243547	lab	N
**MEAN CORP. VOLUME**	0.62	0.642798	1.901794	7.13E-58	lab	N
**RBC BLOOD CELL**	0.6	-0.04311	0.957802	0.188481	lab	N
**ALK PHOSPHATASE, BLD**	0.6	0.308066	1.36079	2.31E-72	lab	N
**MEAN CORP. HGB CONC.**	0.59	-0.05921	0.942505	0.067575	lab	N
**HEMOGLOBIN**	0.58	-0.06242	0.939488	0.039214	lab	Y
**fibroids in pregnancy**	0.55	0.018031	1.018195	1.87E-09	dx	N
**Administrative/social admission**	0.55	0.01568	1.015803	3.51E-33	dx	N
**Hispanic**	0.54	0.401334	1.493817	2.38E-07	demo	N
**twin pregnancy**	0.52	0.015845	1.015971	2.59E-06	dx	N
**Benign neoplasm of uterus**	0.5	0.020208	1.020414	1.31E-10	dx	N
**Other screening for suspected conditions (not mental disorders or infectious disease)**	0.5	0.010497	1.010552	8.35E-15	dx	N
**ALT(SGPT)**	0.49	0.220039	1.246125	9.43E-14	lab	N
**large for GA**	0.48	0.008952	1.008992	0.000193	dx	N
**advanced maternal age**	0.48	0.005319	1.005333	0.00114	dx	Y

Supplementary Table 15: Selected unique features for model week 37

Source feature	Feature importance	Estimate	Adjusted odds ratio	p-value	type	Known associations
**SYSTOLIC BLOOD PRESSURE**	3.73	0.666797	1.947989	9.73E-50	vital	Y
**Other pregnancy and delivery including normal**	2.58	-0.00514	0.994869	0.002743	dx	N
**pe_hist**	1.88	2.468928	11.80978	2.34E-97	demo	Y
**U-PROTEIN**	1.73	0.660369	1.935506	1.3E-226	lab	Y
**age_preg**	1.62	-0.05363	0.947787	0.150268	demo	Y
**URIC ACID-BLD**	1.51	0.601714	1.825245	0	lab	N
**WEIGHT**	1.47	0.053937	1.055418	0.054358	vital	Y
**FIBRINOGEN**	1.42	0.660258	1.935292	0	lab	N
**Headache; including migraine**	1.02	0.031491	1.031992	2.25E-54	dx	Y
**DIASTOLIC BLOOD PRESSURE**	0.96	0.464239	1.590803	1.85E-67	vital	Y
**chronic hypertension**	0.95	0.053067	1.0545	2.3E-143	dx	Y
**PULSE**	0.89	-0.07208	0.93046	0.042221	vital	N
**PLATELET**	0.83	-0.01236	0.987716	0.526013	lab	N
**gestational hypertension**	0.76	0.049682	1.050936	3.1E-239	dx	Y
**Abdominal pain**	0.75	0.011436	1.011502	1.35E-09	dx	N
**advanced maternal age**	0.71	0.00313	1.003135	0.102355	dx	Y
**MEAN CORP. HGB**	0.7	-0.05766	0.943969	0.101504	lab	N
**WHITE BLOOD CELL**	0.69	0.028661	1.029075	0.400537	lab	Y
**RESPIRATIONS**	0.69	0.005019	1.005032	0.891336	vital	N
**MONOCYTE %**	0.69	0.399477	1.491045	5.29E-29	lab	N
**MEAN PLT VOLUME**	0.67	0.668525	1.951358	2.75E-51	lab	N
**RED DISTRIB. WIDTH**	0.63	-0.02714	0.973228	0.455204	lab	N
**intrauterine fetal demise**	0.62	0.016151	1.016282	1.01E-33	dx	N
**obesity In pregnancy**	0.61	0.029116	1.029544	3.26E-33	dx	Y
**MEAN CORP. HGB CONC.**	0.6	-0.16615	0.846915	9.33E-07	lab	N
**EOSINOPHIL %**	0.57	0.151728	1.163843	9.44E-10	lab	N
**PH - DIPSTICK**	0.56	0.032889	1.033436	0.379646	lab	N
**TEMPERATURE**	0.56	0.555667	1.743103	3.13E-54	vital	N
**MEAN CORP. VOLUME**	0.53	0.065907	1.068128	0.092707	lab	N
**HEMATOCRIT**	0.52	-0.0418	0.959065	0.259214	lab	N
**Hispanic**	0.51	0.457163	1.579586	1.64E-07	demo	N
**HEMOGLOBIN**	0.51	-0.02217	0.978077	0.560193	lab	Y
**HEIGHT**	0.51	0.425388	1.530184	1.93E-28	vital	N
**RBC BLOOD CELL**	0.48	0.212174	1.236363	1.86E-11	lab	N
**Other screening for suspected conditions (not mental disorders or infectious disease)**	0.47	0.00997	1.01002	6.99E-11	dx	N
**ALT(SGPT)**	0.45	0.262853	1.300635	4.05E-36	lab	N
**Residual codes; unclassified**	0.45	0.007821	1.007851	8.66E-09	dx	N

Supplementary Table 16: Selected unique features for model week 38

Source feature	Feature importance	Estimate	Adjusted odds ratio	p-value	type	Known associations
**SYSTOLIC BLOOD PRESSURE**	1.77	0.708632	2.031211	5.54E-42	vital	Y
**Other pregnancy and delivery including normal**	1.46	-0.00553	0.994489	0.006918	dx	N
**WEIGHT**	1.39	0.591225	1.806199	5.63E-42	vital	Y
**pe_hist**	1.13	2.397544	10.99614	1.54E-64	demo	Y
**Headache; including migraine**	1.07	0.033321	1.033883	5.78E-53	dx	Y
**age_preg**	1.06	-0.08337	0.920008	0.052113	demo	Y
**URIC ACID-BLD**	1.02	0.59756	1.817678	4.4E-251	lab	N
**FIBRINOGEN**	0.88	0.656714	1.928445	2.6E-265	lab	N
**PLATELET**	0.78	-0.01152	0.988546	0.898893	lab	N
**TEMPERATURE**	0.76	0.545392	1.725285	6.68E-40	vital	N
**MEAN CORP. HGB CONC.**	0.73	-0.11111	0.894841	0.007002	lab	N
**obesity In pregnancy**	0.71	0.029139	1.029568	4.56E-26	dx	Y
**DIASTOLIC BLOOD PRESSURE**	0.69	0.440462	1.553425	2.73E-41	vital	Y
**RESPIRATIONS**	0.68	0.040801	1.041645	0.180907	vital	N
**HEMATOCRIT**	0.68	-0.08239	0.920917	0.005788	lab	N
**MEAN CORP. HGB**	0.67	-0.20279	0.81645	4.14E-09	lab	N
**MEAN PLT VOLUME**	0.64	0.684791	1.983358	1.77E-40	lab	N
**U-PROTEIN**	0.62	0.644881	1.90576	5.1E-164	lab	Y
**Other circulatory disease**	0.58	0.042533	1.043451	1.83E-71	dx	N
**intrauterine fetal demise**	0.57	0.016831	1.016973	1.62E-28	dx	N
**Hispanic**	0.53	0.417013	1.517423	4.31E-05	demo	N
**nonreassuring fetal status**	0.51	0.003795	1.003802	0.028266	dx	N
**Benign neoplasm of uterus**	0.51	0.021257	1.021484	1.45E-07	dx	N
**Allergic reactions**	0.47	0.017642	1.017799	1.61E-22	dx	N
**MONOCYTE %**	0.47	0.445079	1.560613	3.49E-31	lab	N
**SPEC GRAVITY-DIPSTICK**	0.46	0.008159	1.008193	0.779043	lab	N
**RBC BLOOD CELL**	0.46	0.058162	1.059887	0.176843	lab	N
**Residual codes; unclassified**	0.45	0.009113	1.009155	3.07E-09	dx	N
**Abdominal pain**	0.44	0.010506	1.010562	1.77E-06	dx	N
**ALK PHOSPHATASE, BLD**	0.44	0.331939	1.393667	9.61E-50	lab	N
**PULSE**	0.42	-0.03886	0.961882	0.370665	vital	N
**WHITE BLOOD CELL**	0.42	0.101528	1.106861	0.007926	lab	Y
**fibroids in pregnancy**	0.42	0.018757	1.018934	1.57E-06	dx	N
**GTT 3 HOUR**	0.41	0.154483	1.167054	1.92E-06	lab	N
**HEIGHT**	0.41	0.424744	1.5292	1.02E-21	vital	N

Supplementary Table 17: Selected unique features for model week 39

Source feature	Feature importance	Estimate	Adjusted odds ratio	p-value	type	Known associations
**age_preg**	10.53	-0.11124987	0.894715159	0.04448106	demo	Y
**Other pregnancy and delivery including normal**	9.55	-0.004001481	0.996006514	0.153396547	dx	N
**intrauterine fetal demise**	3.17	0.015588482	1.015710617	1.14008E-15	dx	N
**Residual codes; unclassified**	3.16	0.008988653	1.009029173	5.38562E-06	dx	N
**Other screening for suspected conditions (not mental disorders or infectious disease)**	2.97	0.010421225	1.010475715	3.13878E-06	dx	N
**SYSTOLIC BLOOD PRESSURE**	2.59	0.761641504	2.141789091	2.61591E-29	vital	Y
**Headache; including migraine**	2.39	0.038011568	1.038743249	2.02811E-51	dx	Y
**DIASTOLIC BLOOD PRESSURE**	2.26	-0.164154023	0.848611306	0.001691062	vital	Y
**FIBRINOGEN**	2.26	0.668933134	1.952153523	2.0781E-174	lab	N
**TEMPERATURE**	1.91	0.620172957	1.859249584	9.26509E-32	vital	N
**WEIGHT**	1.78	0.626795642	1.871603673	3.18649E-29	vital	Y
**pe_hist**	1.69	2.328142598	10.25886898	5.99289E-33	demo	Y
**Caucasian**	1.65	-0.695580641	0.498784749	7.41951E-10	demo	N
**nonreassuring fetal status**	1.64	0.001503977	1.001505109	0.522886695	dx	N
**PULSE**	1.56	-0.08296264	0.920385532	0.124683631	vital	N
**MEAN PLT VOLUME**	1.48	0.068216109	1.070596649	0.200320819	lab	N
**high risk pregnancy**	1.45	0.017521674	1.017676079	1.73223E-17	dx	N
**MEAN CORP. HGB**	1.34	-0.050990629	0.950287575	0.330304422	lab	N
**MEAN CORP. VOLUME**	1.3	-0.093044334	0.911153104	0.0532552	lab	N
**U-PROTEIN**	1.27	0.638368735	1.893389739	2.0134E-103	lab	Y
**Other circulatory disease**	1.26	0.042058453	1.042955441	1.07653E-43	dx	N
**RED DISTRIB. WIDTH**	1.25	0.069658883	1.072142393	0.099684345	lab	N
**WHITE BLOOD CELL**	1.23	0.190048181	1.209307861	3.14863E-05	lab	Y
**Allergic reactions**	1.22	0.018829603	1.019007998	4.41412E-18	dx	N
**AST (SGOT)**	1.19	0.297207029	1.346093951	2.06114E-32	lab	N
**URIC ACID-BLD**	1.15	0.595776927	1.814440085	1.1016E-159	lab	N
**MEAN CORP. HGB CONC.**	1.14	-0.037068034	0.963610575	0.506923008	lab	N
**RESPIRATIONS**	1.1	0.068104436	1.070477099	0.038099008	vital	N
**advanced maternal age**	1.06	0.002144445	1.002146746	0.475452172	dx	Y
**Administrative/social admission**	1.04	0.016252014	1.016384796	8.13165E-14	dx	N
**HEIGHT**	1.02	0.436859851	1.547839134	8.91864E-15	vital	N
**fibroids in pregnancy**	1.01	0.018673628	1.01884907	0.000240715	dx	N
**PLATELET**	0.88	-0.014015647	0.986082115	0.746480153	lab	N
**Asian**	0.85	-0.707109076	0.49306756	0.016542086	demo	N
**ALT(SGPT)**	0.84	0.082703322	1.086219504	1.43744E-05	lab	N
**gestational hypertension**	0.82	0.043186052	1.04413214	3.37455E-74	dx	Y
**PH - DIPSTICK**	0.82	0.110267793	1.116577042	0.041730474	lab	N
**O2 SATURATION**	0.8	-0.048512005	0.952645902	0.723358835	vital	N

Supplementary Table 18: Selected unique features for model intrapartum

Source feature	Feature importance	Estimate	Adjusted odds ratio	p_value	type	Known associations
**Caucasian**	0.150207522	-0.612958772	0.541745594	6.54E-20	demo	N
**Oxytocin**	0.129205972	-0.011858392	0.988211642	5.22E-08	rx	N
**nonreassuring fetal status**	0.082771879	-0.011251526	0.985542198	4.97E-11	dx	N
**Ketorolac**	0.062647465	-0.010467449	0.989587144	1.19E-06	rx	N
**Sodium Chloride**	0.06007251	-0.0067654	0.993257434	7.28E-06	rx	N
**Other screening for suspected conditions**	0.055253646	0.003910787	1.003615684	7.10E-06	dx	N
**Umbilical cord complication**	0.053902303	-0.009419243	0.985748419	1.06E-05	dx	N
**Headache; including migraine**	0.052181989	0.010596059	1.011519438	1.23E-16	dx	Y
**Administrative/social admission**	0.052019326	0.002447276	1.002543078	0.00705448	dx	N
**age_preg**	0.048287746	0.012246597	1.012321893	0.00126216	demo	Y
**Prolonged pregnancy**	0.04687838	-0.007938494	0.990637602	1.99E-12	dx	N
**twin pregnancy**	0.044835397	0.021500891	1.022383438	2.89E-48	dx	N
**medicaid**	0.043045196	0.190894935	1.210332282	0.00024241	demo	N
**African-American/Black**	0.037403528	0.24701167	1.280194052	0.00035221	demo	N
**Previous C-section**	0.037216406	-0.932613859	0.441434903	5.67E-28	dx	N
**SYSTOLIC BLOOD PRESSURE**	0.033329919	0.015291335	1.015626565	0.00076121	vital	Y
**MONOCYTE %**	0.032845862	-0.034062866	0.966510742	0.00252293	lab	N
**gestational hypertension**	0.031669927	0.703492293	2.016552473	1.55E-07	dx	Y
**DIASTOLIC BLOOD PRESSURE**	0.025997681	0.023066886	1.023745812	9.55E-13	vital	Y
**Malposition; malpresentation**	0.018668199	-0.009098115	0.991244364	8.61E-08	dx	N
**unspecified PROM**	0.016509628	-0.010849348	0.988297504	1.49E-05	dx	N
**Preterm Labor**	0.015716832	-0.002032654	0.998828785	0.00341067	dx	N
**MEAN PLT VOLUME**	0.01522858	0.04512906	1.048222006	0.00046327	lab	N
**ANTIBODY SCREEN**	0.014878611	-0.15267071	0.858412342	0.00767626	lab	N
**Other pregnancy and delivery including normal**	0.013185698	-0.003935741	0.995952399	0.00036824	dx	N
**PULSE**	0.012647335	0.002999201	1.003012165	0.00205978	vital	N
**Medical examination/evaluation**	0.010648062	-0.006882199	0.99433121	2.66E-08	dx	N
**WEIGHT**	0.010487855	0.005728262	1.005758985	1.33E-08	vital	Y
**Other circulatory disease**	0.009448987	0.011407608	1.011614373	2.91E-13	dx	N
**Preterm Labor with Preterm Delivery**	0.007188055	-0.012197647	0.988094143	3.28E-05	dx	N
**HEMOGLOBIN**	0.007144324	-0.073692059	0.928957714	2.80E-06	lab	Y
**RED DISTRIB. WIDTH**	0.006676338	0.022825689	1.023314838	0.00129041	lab	N
**MEAN CORP. HGB CONC**	0.006121853	-0.044000029	0.958026098	0.00273486	lab	N
**WHITE BLOOD CELL**	0.005914148	0.004915298	1.004963856	0.00197973	lab	Y
**obesity**	0.005743344	0.009380888	1.009753816	8.77E-08	dx	Y
**HCT**	0.005470189	-0.001914319	0.998240088	0.00134388	lab	N
**chronic hypertension**	0.005381996	0.00845751	1.00873357	2.31E-06	dx	Y
**MEAN CORP. VOLUME**	0.005217996	-0.007945377	0.992114114	4.77E-05	lab	N
**RBC**	0.004854966	0.100828574	1.110239501	0.00025417	lab	N
**nonspecific fetal complication**	0.00394182	0.003178472	1.003463003	5.64E-05	dx	N
**PLATELET**	0.003940515	-0.007869401	0.993626989	0.00473409	lab	N
**Coagulation and hemorrhagic disorders**	0.003582716	0.006156422	1.006716909	0.00023949	dx	N
**placenta_previa**	0.003204361	-0.007139863	0.992814055	0.00136125	dx	N
**MEAN CORP. HGB**	0.003196592	-0.018455737	0.981936044	0.00094464	lab	N
**anemia in pregnancy**	0.003063244	-0.005399402	0.994080684	0.00011376	dx	N
**gbs infection**	0.002883183	-0.006020683	0.993672105	0.00292173	dx	N
**Hispanic**	0.002823367	-0.096039099	0.908428496	0.17206322	demo	N
**first_drink_impact_day**	0.002415315	0.119240824	1.126641208	0.00413774	demo	N

Supplementary Table 18: Selected unique features for model intrapartum (continued)

Source feature	Feature importance	Estimate	Adjusted odds ratio	p_value	type	Known associations
**Acetaminophen**	0.002231738	0.006407242	1.006427812	1.10E-11	rx	N
**oligohydramnios**	0.002125025	-0.008065892	0.991675763	0.0038694	dx	N
**Asian**	0.001544958	-0.527018632	0.590362436	1.77E-08	demo	N
**renal complications in pregnancy**	0.001482591	0.008358099	1.008117008	0.00104707	dx	N
**Postpartum diagnosis**	0.0012415	-0.024619059	0.975461955	0.00153584	dx	N
**Essential hypertension**	0.001020357	0.006664701	1.007944736	0.00105849	dx	Y
**pe_hist**	0.000660588	1.086209176	2.963020466	3.08E-09	demo	Y
**Labetalol**	0.000504375	0.038119603	1.038855475	1.82E-40	rx	N
**delivery_failed induction of labor**	0	-0.011060667	0.99600403	0.00720967	dx	N
**pregestational type 1 DM**	0	0.019578839	1.023268976	0.00265496	dx	N
**miscarriage**	0	0.031337137	1.031833315	0.83061889	demo	N
**CASE ABORTED**	0	0.061336028	1.063256138	0.0148516	proc	N
**Acute bronchitis**	0	0.019643993	1.01719014	0.00313406	dx	N
**Ligation or transection of fallopian tube(s) when done at the time of cesarean delivery or intra-abdominal surgery (not a separate procedure) (List separately in addition to code for primary procedure)**	0	-0.014728689	0.985379248	0.01244612	proc	N
**headache**	0	0.014726792	1.010664709	0.00504439	dx	Y
**in vitro fertilization**	0	0.006870006	1.005935432	0.00569769	dx	N
**Complications of surgical procedures or medical care**	0	0.008629529	1.008944915	0.00746718	dx	N
**recurrent pregnancy loss**	0	0.010096693	1.009445444	0.00543719	dx	N
**first_smk_impact_day**	0	0.185427649	1.203733105	0.00315571	demo	N
**delivery_hematoma**	0	0.032491191	1.029803666	0.00624365	dx	N

Supplementary Table 19: Selected unique features for model postpartum

Source feature	Feature importance	Estimate	Adjusted odds ratio	p_value	type	Known associations
**Ibuprofen**	0.364716606	-0.01296254	0.987121112	1.04E-30	rx	N
**Caucasian**	0.240070946	-0.397921519	0.671714743	0.022826759	demo	N
**OB-related trauma to perineum and vulva**	0.213517372	-0.013542605	0.985265174	3.97E-21	dx	N
**age_preg**	0.17398007	0.038154054	1.038891266	4.30E-05	demo	Y
**Other aftercare**	0.154464294	-0.01818284	0.981693531	2.45E-15	dx	N
**Administrative/social admission**	0.137355629	0.005672244	1.007675073	0.001819962	dx	N
**Contraceptive and procreative management**	0.128979895	-0.009443669	0.990932444	8.88E-07	dx	N
**Umbilical cord complication**	0.104475754	-0.005182352	0.994909965	0.0002134	dx	N
**gestational hypertension**	0.101781649	0.018124124	1.018196444	1.90E-28	dx	Y
**African-American/Black**	0.099505476	0.850423852	2.340638725	8.31E-07	demo	N
**Immunizations and screening for infectious diseases**	0.087812433	-0.006912188	0.993499583	3.02E-06	dx	N
**SYSTOLIC BLOOD PRESSURE**	0.06972831	0.017756126	1.01830803	0.001350675	vital	Y
**PULSE**	0.059997127	0.010106951	1.010203362	0.001135782	vital	N
**Medical examination/evaluation**	0.059511343	-0.00557685	0.99311459	0.006598619	dx	N
**Headache; including migraine**	0.0583503	0.006450628	1.008291684	0.00545399	dx	Y
**DIASTOLIC BLOOD PRESSURE**	0.054403084	0.01945539	1.020098368	0.000130308	vital	Y
**MEAN CORP. VOLUME**	0.053784586	-0.008597857	0.99146911	0.007918128	lab	N
**WHITE BLOOD CELL**	0.046290387	-0.000641479	0.999447766	0.003521754	lab	Y
**TEMPERATURE**	0.045716487	0.219254459	1.29755792	0.001654393	vital	N
**unspecified fluid disorder**	0.042850071	-0.010161876	0.990712458	0.000346916	dx	N
**Allergic reactions**	0.040199987	-0.005135422	0.994413624	0.000179517	dx	N
**HCT**	0.039177449	-0.026837951	0.973623319	0.003051283	lab	N
**HEMOGLOBIN**	0.033595806	-0.088121674	0.917055952	0.002901927	lab	Y
**MEAN CORP. HGB**	0.032618618	-0.0396916	0.961085793	0.008396058	lab	N
**medicaid**	0.031659207	0.159002897	1.172341343	0.187813075	demo	N
**RESPIRATIONS**	0.028930863	0.044547228	1.046981541	0.001144948	vital	N
**chronic hypertension**	0.027141798	0.008299004	1.006927645	0.001890334	dx	Y
**WEIGHT**	0.025493696	0.006233304	1.006271732	0.001307854	vital	Y
**Residual codes; unclassified**	0.025284996	-0.004883181	0.996869887	0.000813826	dx	N
**decreased fetal movement**	0.024260535	0.006941224	1.007660168	0.000256835	dx	N
**Hispanic**	0.015932268	0.356425473	1.428215086	0.047964851	demo	N
**Labetalol**	0.010603545	0.029787345	1.030235426	2.88E-33	rx	N
**mental health**	0.010160836	0.01181943	1.012007109	0.001298682	dx	N
**pe_hist**	0.008054557	1.898771907	6.677688583	2.02E-11	demo	Y
**twin pregnancy**	0.006906508	0.011611433	1.012797965	1.12E-05	dx	N
**Other circulatory disease**	0.006237221	0.006950202	1.007174409	0.00536639	dx	N
**first_drink_impact_day**	0.003864094	0.272437872	1.313161872	0.003097715	demo	N
**Other endocrine disorders**	0.002160961	0.01208437	1.010729811	0.000606789	dx	N
**Hypertension with complications and secondary hypertension**	0.00192391	0.021280143	1.019747704	0.000317305	dx	Y
**first_smk_impact_day**	0.001369617	0.049109645	1.050335508	0.72639165	demo	N
**Laparoscopy, surgical; cholecystectomy**	0.000887704	0.037601009	1.038316871	0.028805294	proc	N
**Endocervical curettage (not done as part of a dilation and curettage)**	0.000733898	0.03674391	1.037427312	0.021154699	proc	N
**Laparoscopy, surgical, repair, ventral, umbilical, spigelian or epigastric hernia (includes mesh insertion, when performed)**	0	0.04337429	1.044328703	0.015733315	proc	N

Supplementary Table 19: Selected unique features for model postpartum (continued)

Source feature	Feature importance	Estimate	Adjusted odds ratio	p_value	type	Known associations
**Total abdominal hysterectomy (corpus and cervix), with or without removal of tube(s), with or without removal of ovary(s)**	0	0.020873501	1.021092877	0.023667936	proc	N
**Laparoscopic treatment of ectopic pregnancy; without salpingectomy and/or oophorectomy**	0	0.134467869	1.143927902	0.030601679	proc	N
**Asian**	0	-0.062982727	0.938959692	0.782407711	demo	N
**hellp syndrome (severe preeclampsia that affects liver, platelets, hemolysis)**	0	0.060868247	1.054113349	8.58E-10	dx	N
**miscarriage**	0	-0.055010964	0.94647477	0.848525972	demo	N

Supplementary Table 20: Metrics for MSH training dataset

week	ACOG (high risk factors) AUC	ACOG (all risk factors) AUC	SEN	SPE	F1 score	ACC	PPV	NPV	AUC	AP
**4**	0.621 [0.617, 0.626]	0.671 [0.666, 0.677]	0.538 [0.523, 0.562]	0.752 [0.743, 0.762]	0.066 [0.065, 0.068]	0.749 [0.739, 0.758]	0.035 [0.034, 0.036]	0.990 [0.989, 0.990]	0.688 [0.680, 0.696]	0.081 [0.064, 0.096]
**8**			0.534 [0.533, 0.575]	0.790 [0.788, 0.794]	0.079 [0.077, 0.081]	0.787 [0.784, 0.789]	0.042 [0.042, 0.043]	0.990 [0.990, 0.991]	0.707 [0.694, 0.732]	0.099 [0.060, 0.117]
**12**			0.528 [0.493, 0.534]	0.845 [0.828, 0.855]	0.094 [0.090, 0.098]	0.839 [0.824, 0.848]	0.052 [0.049, 0.054]	0.991 [0.990, 0.991]	0.739 [0.730, 0.750]	0.091 [0.079, 0.113]
**16**			0.533 [0.503, 0.569]	0.844 [0.837, 0.855]	0.100 [0.099, 0.104]	0.839 [0.833, 0.849]	0.055 [0.054, 0.058]	0.991 [0.990, 0.992]	0.747 [0.742, 0.763]	0.096 [0.096, 0.106]
**20**			0.551 [0.496, 0.562]	0.856 [0.843, 0.864]	0.103 [0.098, 0.109]	0.850 [0.839, 0.858]	0.057 [0.054, 0.062]	0.991 [0.990, 0.992]	0.764 [0.758, 0.777]	0.103 [0.090, 0.114]
**22**			0.534 [0.511, 0.597]	0.857 [0.840, 0.867]	0.110 [0.098, 0.117]	0.852 [0.836, 0.862]	0.061 [0.054, 0.065]	0.991 [0.990, 0.992]	0.765 [0.762, 0.777]	0.105 [0.081, 0.142]
**24**			0.547 [0.518, 0.575]	0.863 [0.855, 0.875]	0.114 [0.103, 0.121]	0.859 [0.850, 0.869]	0.063 [0.057, 0.068]	0.991 [0.991, 0.992]	0.775 [0.770, 0.783]	0.114 [0.109, 0.145]
**26**			0.568 [0.529, 0.584]	0.863 [0.854, 0.873]	0.118 [0.115, 0.126]	0.858 [0.850, 0.868]	0.065 [0.064, 0.071]	0.992 [0.991, 0.992]	0.786 [0.781, 0.800]	0.141 [0.118, 0.149]
**28**			0.583 [0.514, 0.626]	0.888 [0.879, 0.898]	0.137 [0.130, 0.151]	0.883 [0.873, 0.894]	0.079 [0.073, 0.085]	0.992 [0.991, 0.993]	0.814 [0.786, 0.838]	0.148 [0.128, 0.189]
**30**			0.577 [0.572, 0.610]	0.887 [0.882, 0.902]	0.149 [0.127, 0.158]	0.883 [0.878, 0.896]	0.084 [0.071, 0.091]	0.992 [0.992, 0.993]	0.820 [0.805, 0.831]	0.186 [0.150, 0.200]
**32**			0.594 [0.561, 0.623]	0.911 [0.908, 0.920]	0.165 [0.160, 0.183]	0.907 [0.904, 0.914]	0.096 [0.092, 0.106]	0.993 [0.992, 0.994]	0.835 [0.824, 0.851]	0.214 [0.199, 0.243]
**34**			0.666 [0.586, 0.728]	0.925 [0.919, 0.936]	0.197 [0.174, 0.218]	0.922 [0.914, 0.931]	0.116 [0.100, 0.131]	0.995 [0.993, 0.996]	0.852 [0.848, 0.868]	0.234 [0.185, 0.316]
**35**			0.701 [0.666, 0.727]	0.933 [0.929, 0.943]	0.219 [0.186, 0.232]	0.929 [0.926, 0.941]	0.130 [0.110, 0.139]	0.996 [0.995, 0.996]	0.869 [0.864, 0.907]	0.242 [0.197, 0.284]
**36**			0.768 [0.714, 0.800]	0.934 [0.929, 0.947]	0.215 [0.195, 0.247]	0.932 [0.927, 0.944]	0.127 [0.112, 0.146]	0.997 [0.996, 0.998]	0.895 [0.884, 0.920]	0.259 [0.232, 0.298]
**37**			0.776 [0.762, 0.809]	0.929 [0.927, 0.943]	0.168 [0.161, 0.183]	0.928 [0.926, 0.941]	0.094 [0.089, 0.104]	0.998 [0.998, 0.998]	0.915 [0.885, 0.924]	0.180 [0.170, 0.197]
**38**			0.775 [0.697, 0.811]	0.936 [0.913, 0.950]	0.141 [0.110, 0.166]	0.934 [0.912, 0.949]	0.077 [0.059, 0.093]	0.998 [0.998, 0.999]	0.906 [0.889, 0.922]	0.113 [0.078, 0.140]
**39**			0.697 [0.617, 0.816]	0.951 [0.942, 0.957]	0.100 [0.088, 0.138]	0.948 [0.941, 0.956]	0.053 [0.047, 0.077]	0.998 [0.998, 0.999]	0.898 [0.885, 0.929]	0.076 [0.058, 0.085]
**Intrapartum**	N/A	N/A	0.646 [0.620, 0.650]	0.841 [0.839, 0.843]	0.322 [0.315, 0.328]	0.828 [0.825, 0.830]	0.215 [0.210, 0.220]	0.972 [0.970, 0.972]	0.820 [0.815, 0.825]	0.352 [0.338, 0.361]
**Postpartum**	N/A	N/A	0.530 [0.479, 0.572]	0.951 [0.948, 0.963]	0.195 [0.176, 0.224]	0.946 [0.943, 0.958]	0.118 [0.105, 0.147]	0.994 [0.994, 0.995]	0.893 [0.888, 0.899]	0.194 [0.182, 0.244]

Supplementary Table 21: Preeclampsia prevalence, sample size, number of features and percentage of missing values at different datasets

	Mount Sinai Hospital (MSH) training set				Mount Sinai Hospital (MSH) test set				Mount Sinai West/UW/BI/SL (MSW) test set			
**week**	**prevalence**	**sample size**	**feature #**	**missing %**	**prevalence**	**sample size**	**feature #**	**missing %**	**prevalence**	**sample size**	**number #**	**missing %**
**4**	2.16%	55374	2989	37.58%	1.31%	33379	2989	38.04%	1.57%	8941	2989	37.44%
**8**	2.16%	55344	3057	35.88%	1.31%	33379	3057	36.88%	1.57%	8941	3057	35.17%
**12**	2.16%	55308	3123	34.15%	1.31%	33379	3123	35.67%	1.57%	8941	3123	32.78%
**16**	2.16%	55275	3166	33.28%	1.31%	33351	3166	34.98%	1.57%	8940	3166	31.91%
**20**	2.16%	55210	3203	32.66%	1.32%	33218	3203	34.50%	1.57%	8935	3203	31.38%
**22**	2.17%	55134	3219	32.39%	1.32%	33127	3219	34.29%	1.57%	8925	3219	31.13%
**24**	2.17%	55002	3228	32.19%	1.31%	33021	3228	34.15%	1.57%	8914	3228	30.95%
**26**	2.16%	54838	3244	31.86%	1.29%	32860	3244	33.91%	1.55%	8899	3244	30.58%
**28**	2.15%	54651	3259	31.50%	1.29%	32744	3259	33.66%	1.55%	8882	3259	30.27%
**30**	2.13%	54415	3270	31.22%	1.25%	32595	3270	33.43%	1.56%	8867	3270	30.02%
**32**	2.09%	54040	3291	30.90%	1.19%	32380	3291	33.18%	1.52%	8830	3291	29.75%
**34**	1.98%	53274	3294	30.74%	1.06%	31965	3294	33.05%	1.39%	8765	3294	29.59%
**35**	1.90%	52573	3293	30.70%	0.95%	31581	3293	33.04%	1.32%	8700	3293	29.55%
**36**	1.80%	51171	3268	30.92%	0.89%	30975	3268	33.27%	1.22%	8585	3268	29.75%
**37**	1.48%	47941	3204	31.54%	0.74%	29392	3204	33.86%	1.11%	8267	3204	30.27%
**38**	1.25%	41989	3104	32.60%	0.58%	26161	3104	34.89%	0.77%	7418	3104	31.23%
**39**	1.08%	29064	2903	35.00%	0.51%	19663	2903	37.26%	0.56%	5920	2903	33.44%
**Intra**	6.42%	59148	4136	43.43%	3.51%	34118	4136	44.72%	2.27%	9002	4136	42.38%
**Post**	1.19%	55842	5391	48.44%	0.83%	36520	5391	49.32%	0.58%	8894	5391	46.87%

Supplementary Table 22: Validation performance at MSH test dataset

week	ACOG (high risk factors) AUC	ACOG (all risk factors) AUC	SEN	SPE	F1 score	ACC	PPV	NPV	AUC	AP
**4**			0.440 [0.427, 0.452]	0.821 [0.806, 0.832]	0.062 [0.059, 0.063]	0.816 [0.801, 0.826]	0.033 [0.032, 0.034]	0.990 [0.990, 0.990]	0.658 [0.655, 0.668]	0.048 [0.046, 0.049]
**8**			0.578 [0.565, 0.596]	0.795 [0.769, 0.809]	0.112 [0.106, 0.115]	0.790 [0.766, 0.803]	0.062 [0.058, 0.064]	0.988 [0.987, 0.988]	0.755 [0.749, 0.764]	0.085 [0.083, 0.093]
**12**			0.552 [0.527, 0.562]	0.814 [0.798, 0.829]	0.116 [0.111, 0.122]	0.808 [0.793, 0.823]	0.065 [0.061, 0.069]	0.987 [0.987, 0.988]	0.744 [0.738, 0.746]	0.091 [0.085, 0.095]
**16**			0.580 [0.552, 0.596]	0.797 [0.770, 0.812]	0.113 [0.104, 0.123]	0.791 [0.765, 0.806]	0.063 [0.057, 0.069]	0.987 [0.987, 0.988]	0.741 [0.732, 0.745]	0.106 [0.099, 0.111]
**20**			0.546 [0.517, 0.578]	0.831 [0.792, 0.846]	0.126 [0.114, 0.131]	0.824 [0.787, 0.838]	0.071 [0.063, 0.074]	0.987 [0.987, 0.987]	0.748 [0.742, 0.751]	0.121 [0.108, 0.127]
**22**			0.554 [0.516, 0.596]	0.825 [0.786, 0.854]	0.125 [0.115, 0.133]	0.818 [0.781, 0.846]	0.071 [0.064, 0.077]	0.987 [0.986, 0.988]	0.758 [0.751, 0.759]	0.104 [0.093, 0.116]
**24**			0.565 [0.554, 0.580]	0.828 [0.809, 0.847]	0.129 [0.122, 0.145]	0.821 [0.803, 0.841]	0.073 [0.068, 0.083]	0.987 [0.987, 0.988]	0.761 [0.758, 0.772]	0.120 [0.115, 0.141]
**26**			0.584 [0.564, 0.599]	0.823 [0.811, 0.856]	0.135 [0.129, 0.157]	0.817 [0.806, 0.849]	0.076 [0.073, 0.091]	0.987 [0.987, 0.988]	0.786 [0.776, 0.790]	0.141 [0.134, 0.179]
**28**	0.583 [0.577, 0.589]	0.662 [0.653, 0.672]	0.593 [0.585, 0.612]	0.858 [0.848, 0.877]	0.166 [0.160, 0.179]	0.852 [0.842, 0.870]	0.096 [0.092, 0.106]	0.989 [0.988, 0.989]	0.801 [0.798, 0.805]	0.200 [0.169, 0.208]
**30**			0.619 [0.606, 0.631]	0.867 [0.858, 0.884]	0.176 [0.171, 0.194]	0.861 [0.852, 0.877]	0.103 [0.099, 0.115]	0.989 [0.989, 0.989]	0.815 [0.812, 0.820]	0.251 [0.245, 0.257]
**32**			0.652 [0.634, 0.662]	0.873 [0.852, 0.885]	0.193 [0.167, 0.199]	0.869 [0.847, 0.879]	0.112 [0.096, 0.118]	0.990 [0.990, 0.990]	0.829 [0.825, 0.832]	0.257 [0.236, 0.271]
**34**			0.613 [0.587, 0.630]	0.903 [0.885, 0.917]	0.213 [0.197, 0.239]	0.896 [0.879, 0.909]	0.129 [0.117, 0.148]	0.990 [0.989, 0.990]	0.834 [0.830, 0.837]	0.291 [0.274, 0.320]
**35**			0.620 [0.615, 0.641]	0.912 [0.908, 0.931]	0.223 [0.216, 0.260]	0.905 [0.902, 0.924]	0.136 [0.130, 0.164]	0.991 [0.991, 0.991]	0.852 [0.849, 0.854]	0.277 [0.254, 0.319]
**36**			0.645 [0.633, 0.673]	0.920 [0.913, 0.934]	0.233 [0.224, 0.261]	0.914 [0.908, 0.928]	0.142 [0.135, 0.164]	0.992 [0.992, 0.993]	0.868 [0.862, 0.871]	0.277 [0.232, 0.336]
**37**			0.668 [0.659, 0.682]	0.918 [0.911, 0.929]	0.225 [0.205, 0.239]	0.914 [0.907, 0.924]	0.135 [0.122, 0.146]	0.993 [0.993, 0.994]	0.865 [0.859, 0.870]	0.183 [0.178, 0.215]
**38**			0.615 [0.584, 0.662]	0.923 [0.887, 0.941]	0.162 [0.129, 0.192]	0.919 [0.884, 0.936]	0.094 [0.072, 0.115]	0.995 [0.994, 0.995]	0.844 [0.834, 0.851]	0.126 [0.104, 0.172]
**39**			0.548 [0.540, 0.602]	0.943 [0.920, 0.945]	0.171 [0.150, 0.182]	0.938 [0.916, 0.940]	0.103 [0.086, 0.109]	0.994 [0.994, 0.995]	0.834 [0.824, 0.839]	0.108 [0.092, 0.131]
**Intrapartum**	N/A	N/A	0.444 [0.437, 0.453]	0.925 [0.919, 0.929]	0.268 [0.263, 0.272]	0.907 [0.901, 0.910]	0.194 [0.185, 0.196]	0.976 [0.976, 0.977]	0.833 [0.831, 0.834]	0.208 [0.205, 0.211]
**Postpartum**	N/A	N/A	0.516 [0.491, 0.543]	0.950 [0.941, 0.953]	0.138 [0.126, 0.139]	0.946 [0.937, 0.949]	0.080 [0.071, 0.081]	0.996 [0.996, 0.996]	0.842 [0.841, 0.846]	0.108 [0.105, 0.111]

Supplementary Table 23: Validation performance at MSW test dataset

week	ACOG (high risk factors) AUC	ACOG (all risk factors) AUC	SEN	SPE	F1 score	ACC	PPV	NPV	AUC	AP
**4**			0.486 [0.465, 0.517]	0.772 [0.766, 0.787]	0.063 [0.062, 0.066]	0.768 [0.762, 0.781]	0.034 [0.033, 0.035]	0.989 [0.989, 0.990]	0.675 [0.664, 0.678]	0.045 [0.042, 0.049]
**8**			0.617 [0.601, 0.633]	0.769 [0.760, 0.782]	0.079 [0.076, 0.082]	0.766 [0.758, 0.779]	0.042 [0.040, 0.044]	0.992 [0.992, 0.992]	0.769 [0.759, 0.782]	0.058 [0.056, 0.065]
**12**			0.522 [0.491, 0.539]	0.820 [0.801, 0.835]	0.089 [0.087, 0.096]	0.815 [0.797, 0.829]	0.049 [0.047, 0.053]	0.989 [0.989, 0.990]	0.747 [0.746, 0.758]	0.062 [0.061, 0.069]
**16**			0.561 [0.530, 0.577]	0.808 [0.793, 0.828]	0.089 [0.084, 0.098]	0.804 [0.788, 0.824]	0.048 [0.045, 0.054]	0.991 [0.990, 0.991]	0.751 [0.738, 0.757]	0.060 [0.057, 0.067]
**20**			0.533 [0.521, 0.590]	0.825 [0.799, 0.840]	0.090 [0.085, 0.096]	0.820 [0.796, 0.835]	0.049 [0.045, 0.053]	0.991 [0.991, 0.991]	0.775 [0.769, 0.778]	0.072 [0.068, 0.076]
**22**			0.574 [0.541, 0.602]	0.795 [0.774, 0.834]	0.088 [0.085, 0.098]	0.791 [0.771, 0.829]	0.048 [0.046, 0.054]	0.991 [0.991, 0.991]	0.777 [0.767, 0.781]	0.069 [0.068, 0.073]
**24**			0.562 [0.554, 0.596]	0.819 [0.799, 0.838]	0.097 [0.090, 0.101]	0.815 [0.795, 0.833]	0.053 [0.049, 0.056]	0.991 [0.991, 0.991]	0.782 [0.773, 0.786]	0.082 [0.079, 0.085]
**26**			0.564 [0.538, 0.580]	0.819 [0.812, 0.836]	0.094 [0.091, 0.103]	0.814 [0.808, 0.832]	0.051 [0.050, 0.057]	0.991 [0.991, 0.991]	0.775 [0.773, 0.787]	0.087 [0.081, 0.094]
**28**	0.575 [0.563, 0.587]	0.640 [0.624, 0.659]	0.570 [0.545, 0.586]	0.842 [0.825, 0.866]	0.108 [0.102, 0.119]	0.837 [0.821, 0.861]	0.060 [0.056, 0.067]	0.991 [0.991, 0.991]	0.800 [0.794, 0.805]	0.121 [0.114, 0.124]
**30**			0.581 [0.552, 0.603]	0.852 [0.817, 0.866]	0.116 [0.103, 0.123]	0.847 [0.813, 0.861]	0.065 [0.056, 0.069]	0.991 [0.991, 0.991]	0.785 [0.778, 0.790]	0.120 [0.114, 0.123]
**32**			0.564 [0.530, 0.582]	0.861 [0.837, 0.886]	0.125 [0.105, 0.138]	0.856 [0.832, 0.880]	0.070 [0.058, 0.079]	0.991 [0.991, 0.991]	0.797 [0.786, 0.804]	0.141 [0.135, 0.148]
**34**			0.504 [0.476, 0.550]	0.889 [0.860, 0.912]	0.121 [0.107, 0.141]	0.883 [0.855, 0.905]	0.069 [0.059, 0.082]	0.991 [0.991, 0.992]	0.806 [0.801, 0.816]	0.139 [0.128, 0.153]
**35**			0.486 [0.455, 0.514]	0.917 [0.907, 0.945]	0.139 [0.136, 0.180]	0.910 [0.901, 0.937]	0.081 [0.079, 0.112]	0.992 [0.991, 0.992]	0.823 [0.815, 0.827]	0.148 [0.134, 0.169]
**36**			0.515 [0.483, 0.534]	0.921 [0.913, 0.943]	0.144 [0.131, 0.173]	0.915 [0.908, 0.937]	0.083 [0.075, 0.104]	0.993 [0.993, 0.993]	0.825 [0.815, 0.832]	0.124 [0.110, 0.143]
**37**			0.449 [0.351, 0.478]	0.940 [0.936, 0.956]	0.144 [0.131, 0.152]	0.934 [0.930, 0.948]	0.086 [0.083, 0.091]	0.992 [0.991, 0.993]	0.823 [0.817, 0.825]	0.093 [0.090, 0.105]
**38**			0.307 [0.123, 0.496]	0.953 [0.905, 0.976]	0.075 [0.067, 0.104]	0.948 [0.902, 0.969]	0.044 [0.039, 0.070]	0.994 [0.992, 0.995]	0.791 [0.766, 0.799]	0.059 [0.049, 0.062]
**39**			0.141 [0.102, 0.375]	0.983 [0.956, 0.989]	0.078 [0.063, 0.094]	0.978 [0.953, 0.984]	0.049 [0.042, 0.059]	0.995 [0.995, 0.996]	0.799 [0.778, 0.809]	0.039 [0.033, 0.049]
**Intrapartum**	N/A	N/A	0.325 [0.313, 0.345]	0.882 [0.879, 0.885]	0.105 [0.101, 0.106]	0.869 [0.867, 0.872]	0.062 [0.060, 0.064]	0.982 [0.982, 0.983]	0.739 [0.734, 0.743]	0.055 [0.054, 0.058]
**Postpartum**	N/A	N/A	0.596 [0.567, 0.630]	0.942 [0.934, 0.949]	0.101 [0.094, 0.114]	0.940 [0.932, 0.947]	0.057 [0.051, 0.063]	0.998 [0.997, 0.998]	0.896 [0.881, 0.898]	0.095 [0.091, 0.115]

Supplementary Table 24: Comparisons of prediction performances and features with existing models

Study	AUC (IQ or 95%CI)[a]	Maternal characteristics	History of preeclampsia	Biochemical markers	Doppler	Laboratory results	Time-varying model
Our model (week 16)	0.75 (IQ: 0.74-0.76)	✓	✓			✓	✓
Our model (week 34)	0.85 (IQ: 0.84-0.87)	✓	✓			✓	✓
Marić et al, 2020[1] (week 16)	0.79 (0.75-0.83)	✓	✓			✓	
Wright et al, 2019[2] (week 13)	0.83 (0.81-0.84)	✓	✓	✓	✓		
Wright et al, 2015[3] (week 13)	0.756	✓	✓				
Myatt et al, 2012[4] (week 12)	0.73 (0.69-0.77)	✓	✓	✓		✓	
Odibo et al, 2011[5] (week 14)	0.77 (0.63-0.81)	✓	✓	✓	✓		
North et al, 2011[6] (week 21)	0.71	✓					
Yu et al, 2005[7] (week 24)	0.83	✓	✓		✓		
ACOG – all risk factors (week 4)	0.67 (IQ: 0.67-0.68)	✓	✓				

a. All of the AUCs listed are estimated from the training data set provided from the original manuscripts. Specifically, Marić et al. 2020 computed the AUC used 4-fold cross validation repeated 5 times; North et al. 2011 calculated the AUC by using 10-fold cross-validation repeated 10 times. Wright et al. 2015 used 5-fold single cross-validation. The AUCs listed from Wright et al. 2019, Myatt et al. 2012, Odibo et al. 2011, and Yu et al. 2005 were derived from training data using the predictive model fitted to training data without cross-validation.

Supplementary Table 25: Clinical feature characteristics for each patient across pregnancy

Feature type	Overall (std)	prior pregnancy (std)	pregnancy to delivery (std)	Postpartum (std)
**Procedures by CPT4**	1.12 (0.43)	1.09 (0.34)	1.05 (0.24)	1.23 (0.49)
**Diagnosis by ICD9/10**	21.75 (17.16)	10.17 (6.67)	18.62 (14.51)	7.8 (8.48)
**labs by LOINC**	35.24 (19.03)	19.99 (12.33)	32.74 (16.97)	23.24 (17.89)
**Drugs by Name**	10.63 (8.35)	5.63 (4.95)	7.27 (5.58)	4.89 (5.71)
**# of vital measurements**	246.9 (273.76)	97.57 (140.34)	160.13 (192.95)	49.63 (130.3)

Supplementary References

1. Marić, I. *et al.* Early prediction of preeclampsia via machine learning. *Am. J. Obstet. Gynecol. MFM* **2**, 100100 (2020).
2. Wright, D. *et al.* Predictive performance of the competing risk model in screening for preeclampsia. *Am. J. Obstet. Gynecol.* **220**, 199.e1-199.e13 (2019).
3. Wright, D., Syngelaki, A., Akolekar, R., Poon, L. C. & Nicolaides, K. H. Competing risks model in screening for preeclampsia by maternal characteristics and medical history. *Am. J. Obstet. Gynecol.* **213**, 62.e1-62.e10 (2015).
4. Myatt, L. *et al.* First-trimester prediction of preeclampsia in nulliparous women at low risk. *Obstet. Gynecol.* **119**, 1234–1242 (2012).
5. Odibo, A. O. *et al.* First-trimester placental protein 13, PAPP-A, uterine artery Doppler and maternal characteristics in the prediction of pre-eclampsia. *Placenta* **32**, 598–602 (2011).
6. North, R. A. *et al.* Clinical risk prediction for pre-eclampsia in nulliparous women: Development of model in international prospective cohort. *BMJ* **342**, (2011).
7. Yu, C. K. H., Smith, G. C. S., Papageorghiou, A. T., Cacho, A. M. & Nicolaides, K. H. An integrated model for the prediction of pre-eclampsia using maternal factors and uterine artery Doppler velocimetry in unselected low-risk women. *American Journal of Obstetrics and Gynecology* vol. 195 330 (2006).
